# Pragmatic MDR: a metadata repository with bottom-up standardization of medical metadata through reuse

**DOI:** 10.1186/s12911-021-01524-8

**Published:** 2021-05-17

**Authors:** Stefan Hegselmann, Michael Storck, Sophia Gessner, Philipp Neuhaus, Julian Varghese, Philipp Bruland, Alexandra Meidt, Cornelia Mertens, Sarah Riepenhausen, Sonja Baier, Benedikt Stöcker, Jörg Henke, Carsten Oliver Schmidt, Martin Dugas

**Affiliations:** 1 Institute of Medical Informatics, University of Münster, Münster, Germany; 2 University of Applied Sciences Ostwestfalen-Lippe, Lemgo, Germany; 3 Centre for Clinical Trials, University of Münster, Münster, Germany; 4 Institute of Community Medicine, University Medicine of Greifswald, Greifswald, Germany

**Keywords:** Metadata repository, Metadata standardization, Data integration, ISO/IEC 11179

## Abstract

**Background:**

The variety of medical documentation often leads to incompatible data elements that impede data integration between institutions. A common approach to standardize and distribute metadata definitions are ISO/IEC 11179 norm-compliant metadata repositories with top-down standardization. To the best of our knowledge, however, it is not yet common practice to reuse the content of publicly accessible metadata repositories for creation of case report forms or routine documentation. We suggest an alternative concept called pragmatic metadata repository, which enables a community-driven bottom-up approach for agreeing on data collection models. A pragmatic metadata repository collects real-world documentation and considers frequent metadata definitions as high quality with potential for reuse.

**Methods:**

We implemented a pragmatic metadata repository proof of concept application and filled it with medical forms from the Portal of Medical Data Models. We applied this prototype in two use cases to demonstrate its capabilities for reusing metadata: first, integration into a study editor for the suggestion of data elements and, second, metadata synchronization between two institutions. Moreover, we evaluated the emergence of bottom-up standards in the prototype and two medical data managers assessed their quality for 24 medical concepts.

**Results:**

The resulting prototype contained 466,569 unique metadata definitions. Integration into the study editor led to a reuse of 1836 items and item groups. During the metadata synchronization, semantic codes of 4608 data elements were transferred. Our evaluation revealed that for less complex medical concepts weak bottom-up standards could be established. However, more diverse disease-related concepts showed no convergence of data elements due to an enormous heterogeneity of metadata. The survey showed fair agreement (K_alpha_ = 0.50, 95% CI 0.43–0.56) for good item quality of bottom-up standards.

**Conclusions:**

We demonstrated the feasibility of the pragmatic metadata repository concept for medical documentation. Applications of the prototype in two use cases suggest that it facilitates the reuse of data elements. Our evaluation showed that bottom-up standardization based on a large collection of real-world metadata can yield useful results. The proposed concept shall not replace existing top-down approaches, rather it complements them by showing what is commonly used in the community to guide other researchers.

**Supplementary Information:**

The online version contains supplementary material available at 10.1186/s12911-021-01524-8.

## Background

Due to the medical complexity and heterogeneity of data element definitions, an enormous variety of medical documentation exists [1]. This variety often leads to incompatible data elements that impede data integration between different institutions [2]. Standardizing and reusing such metadata definitions has two major advantages. First, it yields harmonized data sets that allow data exchange between institutions [3, 4] and facilitate data analyses, such as multi-site phenotyping [5] or machine learning [6]. Second, medical documentation does not have to be developed from scratch reducing costs [7]. A common approach pursued in past years to facilitate standardization and reuse are so-called metadata repositories (MDR); databases that gather, retain, and disseminate standardized data element definitions [8]. Several implementations based on the ISO/IEC 11179 norm for metadata registries exist [9]. Table 1 summarizes publicly accessible instances for healthcare applications. Existing MDRs usually apply a top-down approach for metadata standardization through an expert committee or another manually controlled procedure [10]. To the best of our knowledge, however, it is not yet common practice to reuse data element definitions for the creation of case report forms or routine documentation from one of the given MDRs.

**Table 1:** Publicly accessible metadata repositories in the healthcare domain

Repository	Created	Data elements	Scope and content
caDSR [14, 15]	2003	70,472	Defines a comprehensive set of standardized metadata descriptors for cancer research data. It contains common data elements from National Cancer Institute offices and partner organizations
CancerGrid [16]	2005–2010	Website not available anymore	A shared catalogue of standard metadata for cancer trials. It contained common data elements from project partners
CoMetaR [17]	2017	1528	A platform for browsing, discussing, and editing metadata for respiratory diseases. It provides metadata concepts and an ontology
MDM Portal [13]	2011	578,299	Online infrastructure to for creating, analyzing, sharing, and reusing medical forms. It contains medical forms curated by medical experts
METeOR	2005	4668	Australia's repository for national metadata standards for the health, community services, and housing assistance sectors. It provides metadata creation tools and contains endorsed standards
Samply.MDR [18]	2015	672–1936	Open-source MDR implementation for managing and publishing metadata in a standardized and reusable way. It is used for different German study registers
USHIK	2006	29,646	Online, publicly accessible registry and repository of healthcare-related metadata, specifications, and standards. It contains information from numerous healthcare-related initiatives

Repositories were identified via manual review and the PubMed search queries “metadata repository” and “metadata registry”. The content of the table was taken from the given citations and the project websites. Note that the definitions of data elements can vary. The data element counts were generated on 3 Mar 2021

caDSR (https://cdebrowser.nci.nih.gov): The number of data elements was determined via a wildcard search with “*”

CancerGrid (https://www.cs.ox.ac.uk/projects/cancergrid)

CoMetaR (https://data.dzl.de/cometar/web): The provided SPARQL query to search items was used without a search term to identify all items. Unique elements starting with “http://data.dzl.de “ were included as data elements

MDM Portal (https://medical-data-models.org): An internal query was used to determine the data elements

METeOR (https://meteor.aihw.gov.au): The advanced search mechanism with item type *Data Element* was used to determine the number of data elements

Samply.MDR (The following project websites were considered: https://mdr.ccp-it.dktk.dkfz.de/view.xhtml?namespace=dktk, https://mdr.miracum.de, https://mdr.osse-register.de, https://mdr.germanbiobanknode.de): The data elements were determined with the search mechanism excluding outdated elements

USHIK (https://ushik.ahrq.gov): The page https://ushik.ahrq.gov/lists/DataElements?system=mdr provided an overview of all data elements

In this work, we suggest an alternative approach called pragmatic metadata repository, which enables a community-driven bottom-up approach for agreeing on standards and facilitates metadata sharing. We define a pragmatic MDR with the following key principles:

1. Based on real-world metadata definitions that were already used for data collections in medical research or routine healthcare
2. Frequency-based scoring of data elements leading to de facto standards
3. Open access to share, query, and reuse content across institutions

In contrast to existing repositories, a pragmatic MDR contains a large collection of real-world metadata definitions from different sources. To obtain this collection, it allows data sharing for everyone, i.e. it is community-driven. When a data element definition is used in many real-world settings, this indicates that this definition was already tested and is well accepted. Moreover, many data sets already exist, which potentially could be compared to data from a newly designed system that adopts such a data element definition. Hence, the pragmatic MDR concept considers frequent metadata definitions as high quality with an increased potential for reuse and scores them higher. To this end, a pragmatic MDR automatically detects equivalent definitions, aggregates them, and only stores a single copy along with its number of occurrences. We call this concept to reflect metadata quality through its frequency in real-world documentation bottom-up standardization [11]. A comparison to this approach might be the practice to assess the relevance of a scientific paper by its number of citations. This pragmatic MDR concept with bottom-up standardizations shall provide more suitable data element definitions for the creation of case report forms or routine documentation than existing MDRs.

The main objective of this work is to carry out a feasibility study for the suggested pragmatic MDR concept by implementing a proof of concept application fulfilling the above key principles. We apply and evaluate this prototype in two different use cases to demonstrate its capabilities for metadata reuse. First, we integrate it into the study form editor ODMEdit [12] as a suggestion mechanism for data elements during the creation of medical documentation. Second, our partners at the University Medicine of Greifswald use the prototype for automatic synchronization of metadata shared in the Portal of Medical Data Models (MDM Portal) [13]. In addition to that, we perform an evaluation of bottom-up standardization in a pragmatic MDR and verify the quality of the derived data element definitions. To this end, two medical data managers evaluate different properties of the top three items for 24 important medical concepts.

## Methods

### Pragmatic MDR proof of concept implementation

**Fig. 1:**
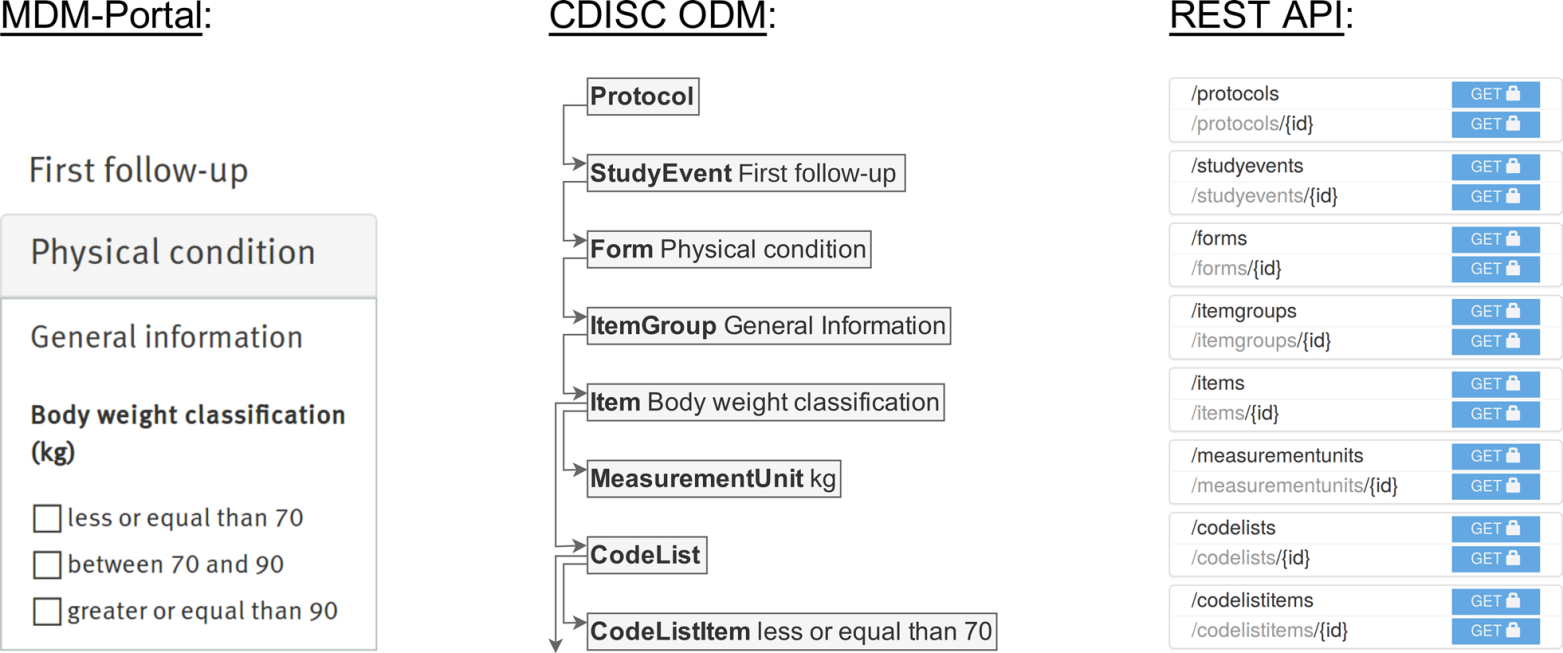
Medical form in the MDM portal with respective ODM elements and REST API endpoints. A medical form with a single data element *Body weight classification* in the MDM Portal (left) with the corresponding ODM definitions as a tree structure (center). Association is indicated with the text behind the ODM definitions. Note that the *Protocol* element is not displayed in the MDM Portal. The right side shows the REST API endpoints of the pragmatic MDR proof of concept implementation to query collections or single resources. The endpoints can be queried with HTTP GET requests and are secured with an API key indicated by the blue fields

**Fig. 2:**
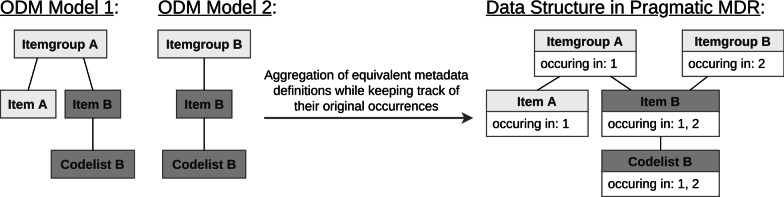
Aggregation of equivalent metadata definitions in the pragmatic MDR proof of concept. Simplified insertion procedure of two ODM models into the pragmatic MDR proof of concept application. Equivalent elements (Item B) and their children (Codelist B) are aggregated while their original occurrences are kept track of. The number of occurrences is used for frequency-based scoring during search

Our implementation was guided by the three principles for a pragmatic MDR. To obtain a large set of real-world metadata definitions for the proof of concept (principle 1), we used the content of the MDM Portal [13]. The portal stores medical forms in the Clinical Data Interchange Standards Consortium (CDISC) Operational Data Model (ODM) [19, 20]. To avoid data conversion and development of a new data model, the prototype’s metadata model was derived from ODM. Figure 1 illustrates ODM’s tree structure in the center with a corresponding example form in the MDM Portal on the left. The eight depicted ODM elements served as atomic resources that were stored in the pragmatic MDR prototype. Sharing of metadata definitions (principle 3) is also realized through the MDM Portal that already offers a simple upload mechanism and automatically synchronizes with the MDR. Our proof of concept implementation split up incoming ODM files into atomic elements and aggregated equivalent elements while it kept a list of their original occurrences as illustrated in Fig. 2. Our prototype treated elements as equivalent if they agreed in every ODM property. We used the open-source search platform Apache Solr [21] with a custom search strategy to rank results with a tradeoff between query matching and the logarithm of their number of occurrences for frequency-based scoring of data elements (principle 2). Open access for querying and reusing metadata definitions (principle 3) was realized through a publicly accessible resource-oriented REST API [22]. For each element, URL endpoints were created to request a single resource or a collection as shown on the right of Fig. 1. We used Spring Boot [23], an open-source Java framework, and PostgreSQL, a database management system, for our implementation.

### Application of pragmatic MDR proof of concept in two use cases

We applied the pragmatic MDR proof of concept in two use cases to demonstrate its capabilities for reusing metadata. The first use case was a suggestion and reuse mechanism for *ItemGroup* and *Item* resources in the study editor ODMEdit [12]. This web-based editor was implemented in R and the suggestion mechanism was realized with JavaScript. To insert a metadata definition into the current working document, ODMEdit processed the JSON response of a specific *ItemGroup* or *Item* resource and transformed it into its internal representation format. The second use case was a collaboration with the University Medicine of Greifswald, which is coordinating the Study of Health in Pomerania (SHIP); a major epidemiological study in Germany initiated in 1997 to obtain scientific valid data regarding factors contributing to a shorter life expectancy in eastern Germany [24]. In prior work, the metadata of SHIP was already converted to ODM and was imported into the MDM Portal [25]. This process included semantic annotation with Unified Medical Language System (UMLS) codes [26] by medical experts. Since this is a laborious process, we wanted to integrate this valuable information into the SHIP database. To this end, we implemented a script that automatically queried all SHIP metadata definitions from the pragmatic MDR and transferred semantic codes into the SHIP data dictionary.

### Evaluation of bottom-up standardization in the pragmatic MDR proof of concept

Our evaluation of bottom-up standardization was two-fold: first, we checked to what extent bottom-up standards emerged in our proof of concept that was filled with the content of the MDM Portal; second, we evaluated the quality of these standards. While the prototype contained different metadata resources, items were used for this evaluation, since they were the smallest building block that is commonly standardized and shared. Moreover, we restricted the evaluation to items with an English question text. To cover a broad spectrum of relevant item definitions for our evaluation, six item concepts were chosen from four different groups: *Clinical Data Acquisition Standards Harmonization *(*CDASH*)* vital signs* [27], six most frequent *Logical Observation Identifiers Names and Codes *(*LOINC*)* codes* [28], items related to *ischaemic heart disease*, and items related to *stroke*. CDASH vital signs and LOINC codes are common data elements used in medical documentation. Ischaemic heart disease and stroke are the top two global causes of death according to the world health organization [29]. For CDASH vital signs and LOINC codes, we used their names to query the pragmatic MDR. To identify important items related to ischaemic heart disease and stroke, we identified relevant medical documentation in the MDM Portal^1^ and collected all medical concepts (UMLS codes) they contained. We then used the UMLS names of the six most frequent concepts for each disease as search queries. The second column in Table 2 shows the resulting 24 queries.

**Table 2:** Overview of item definitions for evaluation of bottom-up standardization

Group	Item concept query	Top three search results	Occ	Top three clustered questions	Occ
CDASH vital signs	Body height (total results: 5363)	Body height	23	Body height	113
		Body height	21	Height	383
		Body height	11	Body height—standing	5
	Body Weight (total results: 6822)	Body weight	20	Body weight	254
		Body weight	40	Current body weight	5
		Body weight	26	Body weight—unit	4
	Diastolic BP (total results: 3052)	BP	21	bp	42
		Diastolic BP	18	Diastolic bp	21
		Semi-supine BP diastolic	10	Semi-supine bp diastolic	10
	Systolic BP (total results: 3373)	BP	21	bp	42
		Systolic BP	18	Systolic bp	22
		Systolic BP	1	Semi-supine bp systolic	10
	Pulse (total results: 1024)	Pulse	28	Pulse	281
		Pulse	21	Pulse	25
		Pulse	19	Pulse rate	50
	Body Temperature (total results: 5806)	Body temperature	22	Body temperature	75
		Body temperature	10	Body temperature unknown at admission	1
		Body temperature	9	Body temperature (c f)	2
Most frequent LOINC codes	Creatinine (total results: 3878)	Creatinine	22	Creatinine	286
		Creatinine	5	Creatinine clearance	71
		Creatinine	18	Serum creatinine	102
	Hemoglobin (total results: 2495)	Hemoglobin	7	Hemoglobin	278
		Hemoglobin	29	Hemoglobin ctc	17
		Hemoglobin	29	Mean corpuscular hemoglobin concentration	45
	Potassium (total results: 842)	Potassium	32	Potassium	226
		Potassium	25	Potassium units	11
		Potassium	15	Potassium results	11
	Glucose (total results: 2108)	Glucose	32	Glucose	226
		Glucose (Serum)	6	Glucose (serum)	7
		Glucose	16	csf: glucose (csf)	6
	Sodium (total results: 783)	Sodium	32	Sodium	207
		Sodium	32	Sodium measurement	12
		Sodium	15	Sodium units	11
	Urea nitrogen (total results: 758)	Blood urea nitrogen	12	Blood urea nitrogen	50
		Blood urea nitrogen	11	Serum urea	3
		Blood urea nitrogen	10	Serum urea nitrogen	1
Most frequent ischaemic heart disease related UMLS concepts from MDM Portal	Myocardial infarction (total results: 3084)	Myocardial infarction	3	Myocardial infarction	80
		Myocardial infarction	21	Patients must not have had myocardial infarction within 6 months of registration	1
		Myocardial infarction	10	mi	1
	Coronary Artery Bypass Surgery (total results: 8781)	CABG	1	cabg	5
		Coronary artery bypass surgery (CABG-Op)	1	coronary artery bypass surgery (cabg-op)	1
		Coronary Artery Bypass Surgery	2	Coronary artery bypass surgery	8
	Angina Pectoris (total results: 1477)	Angina pectoris	8	Angina pectoris	23
		Unstable angina pectoris	14	Unstable angina pectoris	15
		Angina	1	Angina	7
	Myocardial Ischemia (total results: 3112)	Myocardial ischemia	1	Myocardial ischemia	1
		Acute myocardial ischemia/myocardial infarction	1	Acute myocardial ischemia/myocardial infarction	1
		Evidence of myocardial ischemia	1	Evidence of myocardial ischemia	1
	Coronary heart disease (total results: 29,576)	Coronary heart disease	5	Coronary heart disease	12
		Any contraindication to the use of Adrenaline	1	Any contraindication to the use of adrenaline	1
		Coronary Artery Disease (heart disease)	2	Coronary artery disease (heart disease)	2
	Coronary revascularization (total results: 3016)	[4] Non-coronary revascularisation	1	[4] non-coronary revascularisation	1
		4. Has the subject undergone a coronary revascularisation since the last visit?	1	4. Has the subject undergone a coronary revascularisation since the last visit?	1
		Date of revascularisation percutaneous coronary intervention	1	Date of revascularisation percutaneous coronary intervention	1
Most frequent stroke related UMLS concepts from MDM Portal	Cerebrovascular accident (total results: 1376)	Cerebrovascular accident	21	Cerebrovascular accident	27
		Cerebrovascular accident within 1 year	1	Cerebrovascular accident within 1 year	1
		Cerebrovascular disease	1	Cerebrovascular disease	9
	Hemorrhage (total results: 904)	Is a vitreous hemorrhage present?	38	Is a vitreous hemorrhage present?	38
		Hemorrhage	1	Hemorrhage	13
		Vitreous Hemorrhage	4	Vitreous hemorrhage	8
	Transient Ischemic Attack (total results: 1348)	History of transient ischemic attack (TIA)	2	History of transient ischemic attack (tia)	3
		Stroke/transient ischemic attack (TIA)	1	Stroke/transient ischemic attack (tia)	1
		Transient Ischemic Attack	21	Transient ischemic attack	28
	Muscle Weakness (total results: 948)	Muscle weakness	2	Muscle weakness	2
		39. Muscle weakness	1	39. Muscle weakness	1
		Musculoskeletal Muscle atrophy or weakness	1	Musculoskeletal muscle atrophy or weakness	1
	Grip strength test left hand (total results: 15,426)	Left Grip Strength Max-Grip Test 1	2	Left grip strength max-grip test 1	4
		Left Grip Strength Max-Grip Test 1	2	Left grip strength max-grip test 2	4
		Left Grip Strength Max-Grip Test 2	2	Right grip strength max-grip test 1	4
	Dysarthria (total results: 120)	Dysarthria	2	Dysarthria	9
		Dysarthria	4	Severe dysarthria	2
		Dysarthria	1	14. Dysarthria	1

For the evaluation of bottom-up standardization in the pragmatic MDR proof of concept, we used search queries for six different medical concepts (column 2) taken from four different groups (column 1). The entries in column 2 were used to query the pragmatic MDR and the resulting top three search results ranked by frequency-based scoring along with their number of occurrences (Occ) were determined (columns 3 and 4). The last two columns show the same results for a relaxed equivalence definition that only required the ODM question element in lower case to coincide

To evaluate whether bottom-up standards emerged, we plotted cumulative occurrences of item definitions for each search query. Moreover, we determined the ratio of occurrences of the top three search results compared to all results of a query. The top three search results should make up a considerable amount of all items to be considered as standards. Since our prototype implementation required item definitions to agree in every property to aggregate them, we expected this ratio to be low. A first experiment confirmed this suspicion. Hence, we performed the same analyses with a relaxed equivalence definition that only required the ODM question element in lower case to coincide. We chose the question element because this is the text displayed to users. Since the search mechanism also considered partial matches, this analysis might have included item definitions that were only slightly related to the original medical concept, especially when the search query consisted of several words. Nevertheless, we thought that this analysis could yield insights into the emergence of bottom-up standards. Note that we performed this evaluation 1 year after the initial synchronization, so it was based on a larger amount of content from the MDM Portal.

In the second part of our evaluation, two medical data managers evaluated the quality of the top three bottom-up standards derived for each medical concept query. We selected the top three results for evaluation to have a larger set of test samples. The evaluation was performed with a self-designed questionnaire, which included questions for eight ODM item properties: *Question*, *CodeList*, *Name*, *DataType*, *Length*, *Description*, *Alias*, *RangeCheck*. Questions were derived from the definitions in the ODM standard [19]. Moreover, the data managers assessed whether the identified item definitions were a good match for the search query and their relevance for reuse in a case report form. This resulted in ten questions for each item definition. Rating was performed with an ordinal Likert Scale from one to five: *strongly disagree* (*SD*), *disagree* (*D*), *neither agree nor disagree* (*N*), *agree* (*A*), *strongly agree* (*SA*). We did a test evaluation with different item definitions and used the feedback to design the final evaluation questionnaires. We generated descriptive statistics for the ratings of each evaluator and calculated Krippendorff's alpha coefficient with bootstrap confidence intervals as a statistical measure for interrater agreement [30]. All analysis methods were determined a priori in a study protocol. For the final analysis, the color maps and export methods of the heat maps were adjusted slightly to account for correct formatting. Evaluation questionnaires and our study protocol are available as Additional files 1 and 2.

## Results

### Pragmatic MDR proof of concept implementation

**Fig. 3:**
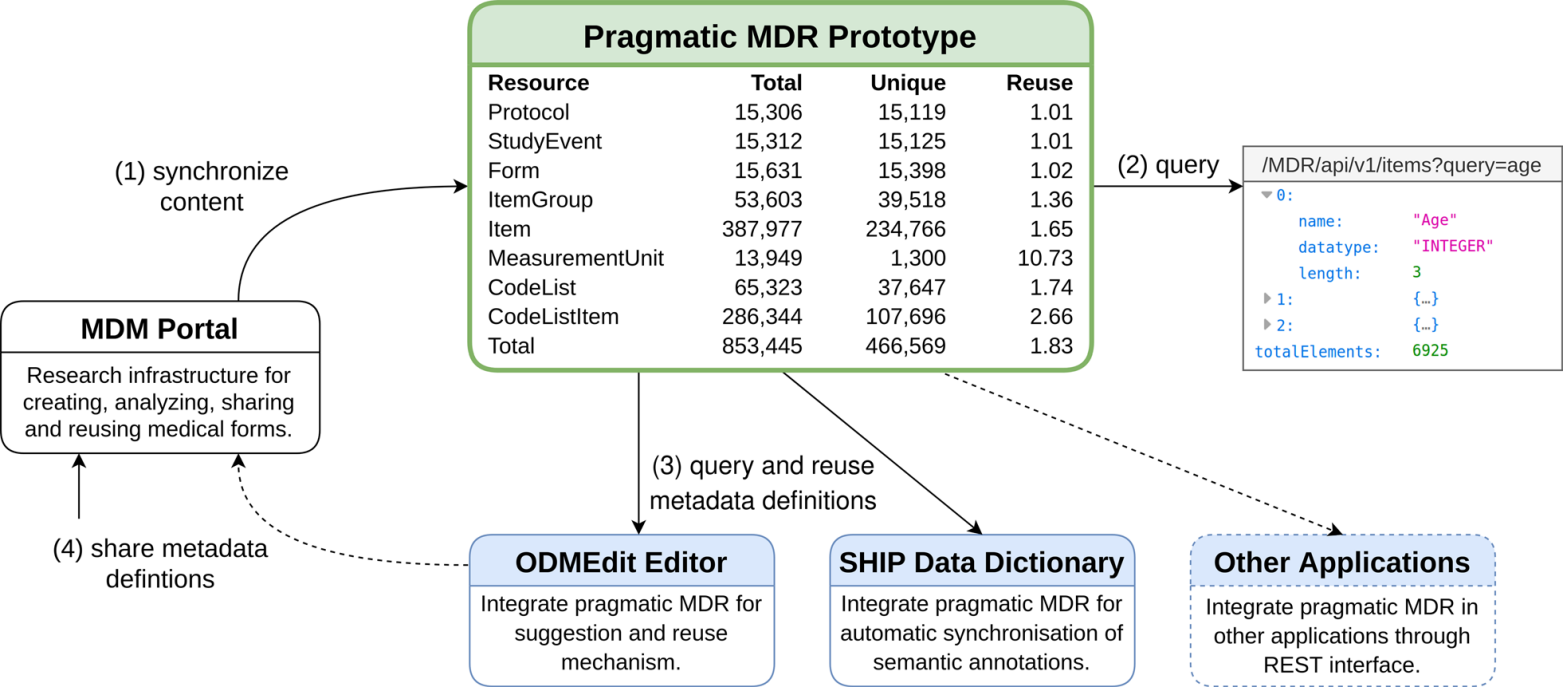
Schematic overview and content of pragmatic MDR proof of concept implementation. This overview shows the content of the pragmatic MDR proof of concept for each ODM element after the initial synchronization with the MDM Portal. New content in the MDM Portal is automatically transferred to the pragmatic MDR (1) and the portal also serves as a frontend to share new metadata definitions (4). The REST API can be queried manually (2) or it can be integrated into applications that query data automatically, e.g. for reuse of metadata definitions (3)

Figure 3 shows a schematic overview of the resulting proof of concept application. Initially, 15,306 medical forms in ODM format were transferred to the pragmatic MDR. New forms that were uploaded to the portal were synchronized automatically (1). Figure 3 contains a table showing total and unique counts for the resulting resources in the pragmatic MDR. There were fewer unique resources because equivalent metadata definitions were aggregated. Reuse indicates the ratio of total and unique resources, i.e. it shows the average number of equivalent definitions. In total, the pragmatic MDR contained 853,445 metadata definitions of which 466,569 were unique. Most resources belonged to the type *Item* and *CodeListItem* with 387,977 and 286,344 elements. Together with *MeasurementUnit* and *CodeList* they had the highest reuse ratio. The REST API can be queried manually and responds in JSON format (2). The depicted example query illustrates a request to the item endpoint with a single parameter *query*.^2^ The main purpose of the API is to enable the integration into applications that query and reuse metadata definitions in an automatic fashion (3). We demonstrated this for the study editor ODMEdit and the SHIP data dictionary. Medical metadata created with these applications or metadata from external sources can be shared via the MDM Portal (4). In this way, a feedback loop is established in which reused metadata definitions are shared again and can contribute to bottom-up standardization.

### Application of pragmatic MDR proof of concept in two use cases

For the first use case, we integrated the pragmatic MDR into the study editor ODMEdit [12] as a suggestion mechanism to explore existing metadata definitions for *ItemGroup* and *Item* resources. Moreover, it was possible to reuse complete definitions and integrate them into the current working document. This allowed to assess and directly reuse 39,518 unique *ItemGroup* and 234,766 *Item* definitions within ODMEdit. Usage statistics showed that 955 *ItemGroup* and 881 *Item* resources were reused during a 9-month test period with medical experts creating medical documentation for the MDM Portal [13]. In the second use case, we integrated the pragmatic MDR prototype into the SHIP data dictionary [24]. A script queried the pragmatic MDR REST API with unique item identifiers to retrieve semantic coding that was added by medical experts in the MDM Portal. During this process, semantic codes were transferred for 4608 data elements. To our knowledge, this is one of the largest efforts to exchange metadata between different institutions in an automatic fashion. Medical experts need on average 1 min to code a single item [31], so this transfer saved approximately 77 h of work.

### Evaluation of bottom-up standardization in the pragmatic MDR proof of concept

We analyzed 24 item concepts from four different categories for our evaluation of bottom-up standardization. The search query for each concept along with the amount of total search results is given in the second column of Table 2. Since the search mechanism also took into account partial matches, though, with a lower score, queries with several words tended to return more results. The third and fourth columns in Table 2 contain the ODM *Question* property and the number of occurrences of the top three search results. Note that the *Question* property could be the same across different items when these data elements differed in other properties (see concept Sodium). Moreover, the search mechanism used a combination of frequency and query matching, hence the first result did not necessarily have the most occurrences (see concept Hemoglobin). In this case, the first item definition was a better match for the search query, which lead to a higher score even though it had fewer occurrences. The last two columns contain the question and occurrences for a relaxed equivalence definition that only required the question texts in lowercase to coincide. Item definitions for the quality evaluation are given in Additional file 4.

**Fig. 4:**
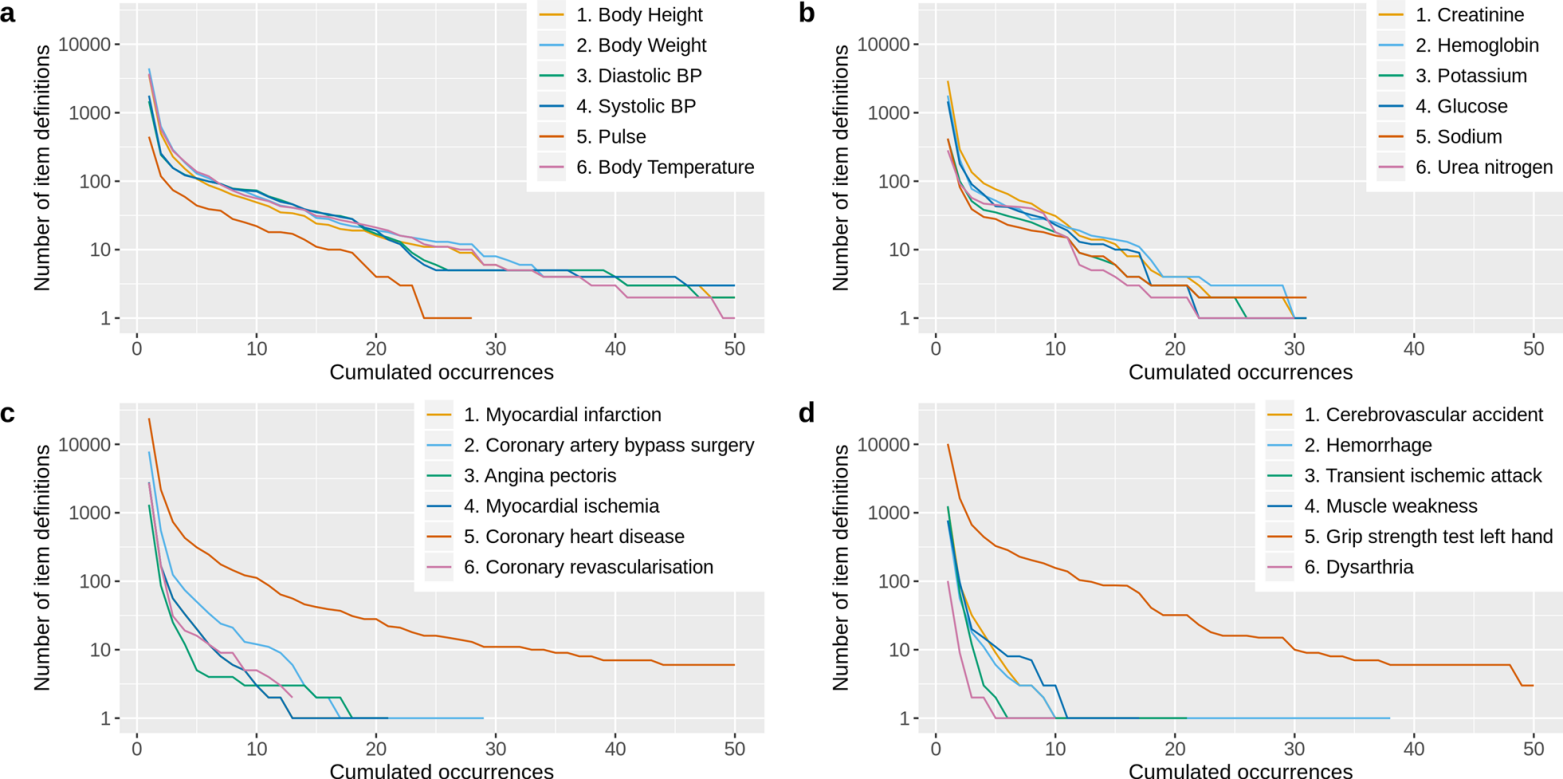
Plots of cumulative occurrences for 24 analyzed item concepts. Cumulative occurrences of all items in the pragmatic MDR proof of concept were generated with the item concept queries in Table 2. Each plot contains six item concepts from one category. **a** CDASH vital signs. **b** Most frequent LOINC codes. **c** Most frequent ischaemic heart disease-related UMLS concepts from MDM Portal. **d** Most frequent stroke-related UMLS concepts from MDM Portal

**Fig. 5:**
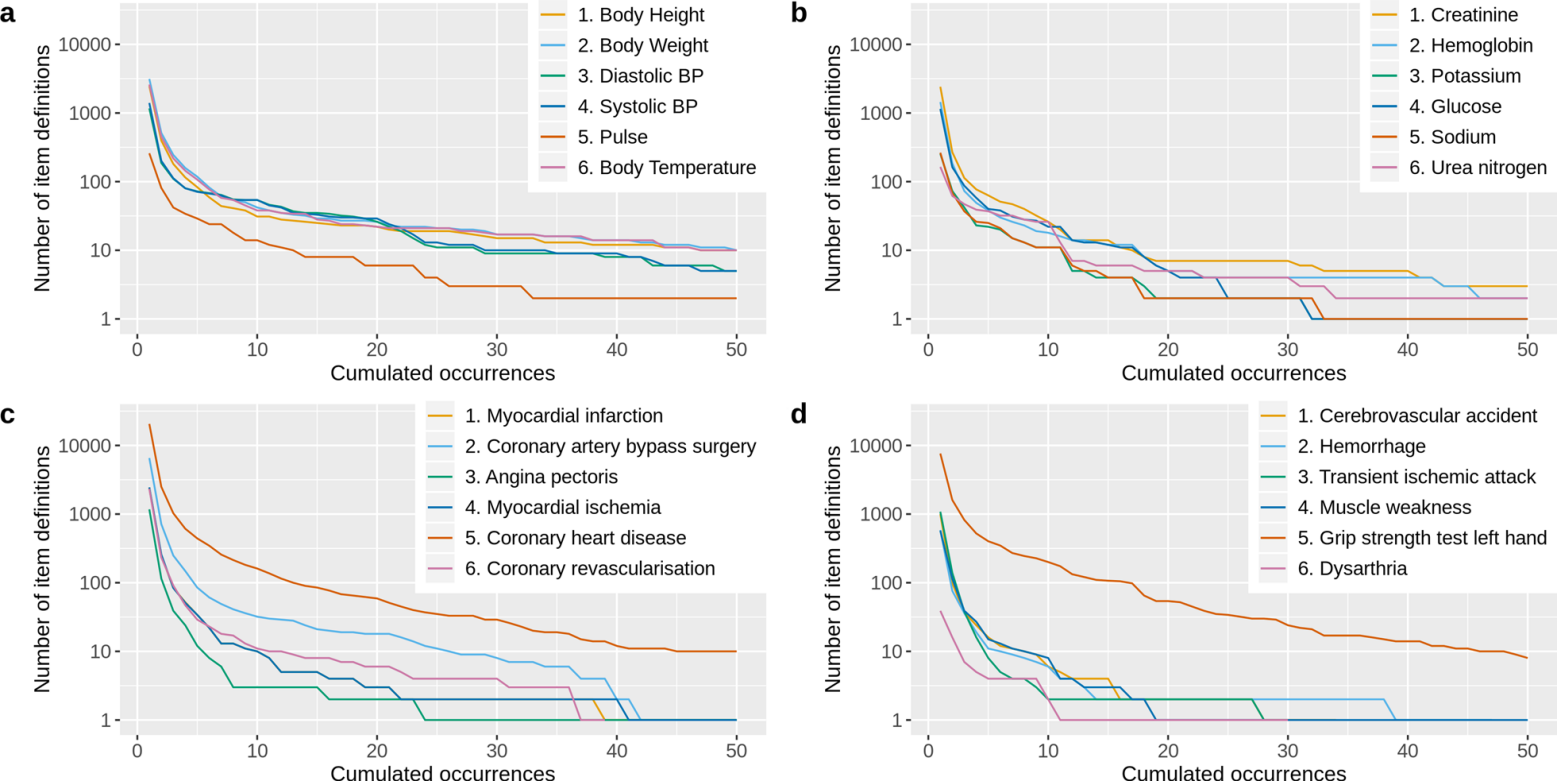
Plots of cumulative occurrences for 24 analyzed item concepts with equivalence on question level. Cumulative occurrences of all items in the pragmatic MDR proof of concept were generated with the item concept queries in Table 2. In contrast to Fig. 4, item concepts were clustered based on their lowercase question text. Each plot contains six item concepts from one category. **a** CDASH vital signs. **b** Most frequent LOINC codes. **c** Most frequent ischaemic heart disease-related UMLS concepts from MDM Portal. **d** Most frequent stroke-related UMLS concepts from MDM Portal

In Fig. 4 cumulative occurrences of the search results for each item concept query are shown. Consider, for example, the item query “Pulse” in plot (a); there was one item definition that occurred 28 times, there were three definitions that occurred at least 23 times, there were 10 definitions that occurred at least 17 times, and 446 definitions that occurred at least once, i.e. in total. This was a common trend across all item concepts. Few item definitions occurred very often, but there were a lot of definitions that occurred only once or twice. For ischaemic heart disease and stroke-related concepts, most frequent definitions had fewer occurrences than CDASH vital signs or LOINC codes. Item concept queries consisting of several words led to many search results and several very frequent item definitions because the search mechanism also included partial matches. Hence, for instance, the query “Coronary heart disease” also returned all item definitions that contained the term “disease”. The ratios of occurrences for the top three search results compared to all results were 2.07 ± 2.06% for CDASH vital signs*,* 4.89 ± 3.30% for LOINC codes, 0.49 ± 0.61% for ischaemic heart disease, and 2.42 ± 2.15% for stroke. We repeated the same analyses with a relaxed equivalence definition that only required the ODM question element in lower case to coincide. Figure 5 shows the plots for cumulative occurrences and they show an increased number of definitions with many occurrences. The ratios of occurrences for the top three results were 8.98 ± 11.83%, 17.13 ± 8.90%, 1.02 ± 1.30%, and 3.68 ± 3.52%. Looking at the absolute number of occurrences in Table 2, we can observe that for CDASH vital signs and LOINC codes most definitions had many occurrences. This effect increased with the relaxed equivalence definition. For ischaemic heart disease and stroke-related concepts, however, there were only a few definitions with many occurrences and the top three results often had only one or few occurrences even when clustered by the question text.

Results of the quality evaluation are summarized in Table 3. It contains an overview of responses for both raters and each item property. Since the ODM standard defines *CodeList*, *Length*, *Description*, *Alias*, and *RangeCheck* as optional attributes, some of these properties were undefined and could not be assessed (column *Undefined*). A single response of rater A for the description property was invalid, which we treated as *Undefined*. The last row summarizes all responses of both raters. Moreover, median values are highlighted in bold. We can observe that *RangeCheck*, *CodeList*, and *Description* properties were missing very often. For *CodeList* this was due to the fact that some items did not offer a value list for selection. On the other hand, only four *Alias* properties were missing indicating a high coverage of semantic codes among top search results. Overall rating of the item definitions was positive (median for both raters and all responses is *A*). Responses of rater A were slightly more positive than of rater B with one median value for *N*, seven for *A*, and three for *SA* compared to one median value for *D*, three for *N*, five for *A*, and one for *SA*. Interrater agreement of both raters could be considered as fair agreement (K_alpha_ = 0.50, 95% CI 0.43–0.56) [32] (Additional file 5 provides a contingency table for rater agreement). Item properties *Question*, *DataType*, and *Alias* were rated higher than *CodeList*, *Description*, and the item’s relevance and differences between item categories, i.e. concepts 1–6, 7–12, 13–17, and 18–24, were very small (see Additional file 6).

**Table 3:** Responses for quality evaluation of bottom-up standards

	Undefined	Rater A					Rater B				
		SD	D	N	A	SA	SD	D	N	A	SA
Question	0	0	3	14	**26**	29	2	4	15	**22**	29
CodeList	56	0	1	5	**6**	4	0	7	**4**	1	4
Name	0	0	3	10	**24**	35	0	16	3	**41**	12
DataType	0	0	6	3	13	**50**	0	8	1	10	**53**
Length	25	0	1	5	**40**	1	0	1	**26**	16	4
Description	49 (50)	0	1	**10**	**9**	2	2	4	**10**	5	2
Alias	4	0	3	5	20	**40**	0	8	8	**40**	12
RangeCheck	71	0	0	0	**1**	0	0	0	0	**1**	0
Good match	0	0	5	11	13	**43**	0	7	15	**43**	7
Relevancy	0	0	4	26	**36**	6	2	**38**	11	21	0
Total	205 (206)	0	27	89	**188**	210	6	94	93	**199**	123

Overview of responses for both raters and item properties. Column *Undefined* shows the number of missing properties that could not be rated. The remaining columns contain the number of responses for *strongly disagree* (SD), *disagree* (D), *neither agree nor disagree* (N), *agree* (A), and *strongly agree* (SA). Median values for each category and rater are highlighted in bold

## Discussion

### Heterogeneity of medical metadata hampers bottom-up standardization

We investigated the concept of bottom-up standardization in a pragmatic MDR that imported at least 15,306 medical forms, identified equivalent definitions, and scored them according to their number of occurrences. To evaluate the emergence of bottom-up standards, we considered 24 important medical concepts and analyzed cumulative occurrences of related items and determined absolute and ratios of occurrences of the top three search results for each concept. However, plots of cumulative occurrences took into account all related items and they were skewed by partial matches of the search mechanism, so we consider them less relevant. Analysis of the top three search results, on the other hand, was more specific, because these item definitions received the best tradeoff between query matching and the number of occurrences. Hence, in the following, we focus on absolute and ratios of occurrences.

For *ischaemic heart disease* and *stroke* concepts, the occurrence ratio and absolute occurrences showed that no clear bottom-up standards emerged in the pragmatic MDR prototype. There were usually more than a thousand total search results, but many of the most frequent definitions only had one or few occurrences. While this effect decreased a little bit with the relaxed equivalence definition using only the question text, we would not call the results clear bottom-up standards. Hence, as a main result of our analysis, we can conclude that there exists an enormous heterogeneity of metadata for medical concepts for diseases. This is consistent with previous work that showed a strong need for metadata harmonization to generate disease-specific common data elements [33, 34].

The situation was different for *CDASH vital signs* and *LOINC codes*. The latter already showed a considerable occurrence ratio with strong item equivalence, which increased to 17.13 ± 8.90% when applying equivalence only on question level. That means for LOINC codes three bottom-up standardized questions represented on average 17.13% of all questions that matched the respective search query. In addition to that, the absolute numbers of occurrences were also very high. Hence, we conclude that for laboratory values our proof of concept was able to determine bottom-up standards. While for CDASH vital signs the occurrence ratios were not as high as for LOINC codes, probably due to higher numbers of total search results, we think the ratio for the relaxed equivalence definition of 8.98 ± 11.83% in combination with a high absolute number of occurrences suggests that weak bottom-up standards emerged.

This discrepancy between the medical categories probably stems from a lower medical complexity: it is easier to agree on data elements to collect vital signs or laboratory values than information on a complex medical condition. However, we are also convinced that for CDASH vital signs and LOINC codes there is still much room for improvement. For instance, consider the concept *Body Temperature* with the relaxed equivalence criteria (Table 2). The second and third search results had only one and two occurrences, which is unlikely to reflect the heterogeneity of collecting the body temperature. Moreover, the bottom-up standards for CDASH vital signs and LOINC codes were very simplistic. There were no complex question texts in the top three search results since it is probably much harder to agree on those.

Our self-designed quality evaluation of bottom-up standards showed an overall fair agreement for good item quality. However, we think this evaluation has only weak validity since there were only two raters and the questions were derived from the ODM standard, which was the format of the original data. Moreover, there might be different use cases for item definitions that were not well reflected in our questionnaire. We conclude that our evaluation gives a hint that our proof of concept can offer useful item definitions for certain scenarios even when bottom-up standards might not emerge.

### A pragmatic MDR can facilitate reusing and sharing of metadata

Reusing medical metadata saves costs in the creation of medical documentation and fosters harmonized data collections [7]. In contrast to existing MDRs, a pragmatic MDR usually offers a larger variety of different metadata definitions for the same medical concept. This allows users to choose a definition from several suggestions, which can facilitate metadata reuse. We demonstrated this for the study editor ODMEdit [12], but also external applications are possible [35].

It is common today that designers of medical information systems do not publish their documentation [36]. Sharing metadata in a pragmatic MDR should only require its occurrence in a real-world data collection; all data processing and bottom-up standardization should be performed automatically. Hence, the sharing process can be simplified to a file upload as we have realized it for our prototype. By reducing the effort to publish medical documentation, a pragmatic MDR might increase the amount of shared metadata. Furthermore, due to the simple policy for metadata sharing, a pragmatic MDR can be used to transfer metadata. We have demonstrated this in the second use case: we reused SHIP metadata even though these definitions did not necessarily emerge as bottom-up standards.

### Bottom-up versus top-down approach for metadata standardization

We discuss some theoretical considerations of bottom-up and top-down standardization not verified in this study to outline key differences and to give an idea where each concept might be advantageous. Bottom-up standards should be the most frequent definitions of a medical concept in a collection of real-world metadata, which usually indicates that they were already used in many settings and are well accepted. Second, since many existing data collections already use this data element, reusing it leads to compatible data collections. Third, bottom-up standardization automatically adapts to changes since shared documentation directly shows up in a pragmatic MDR and influences the scoring mechanism. In addition to that, automatic processing of shared metadata can yield a more neutral scoring of data elements and reduces standardization costs. Lastly, bottom-up standardization offers several candidate definitions, which might be better suited to reflect the heterogeneity of medical documentation. However, this data-driven approach requires a large amount of shared metadata definitions and bottom-up standards highly depend on the data quality. In our feasibility study, we simulated this process with forms from the MDM Portal, which are curated by medical experts. Besides, frequency alone cannot measure the quality of a data element. Due to the large number of different data elements for important medical concepts, many high-quality definitions with few usages will receive a low score and, hence, will be difficult to find in a pragmatic MDR.

The top-down-approach, on the other hand, offers full control to define a single source of truth for data collections, which is necessary to enforce guidelines for semantic interoperability. The quality of these top-down metadata definitions depends on the expertise and opinion of experts. Certainly, real-world examples will be considered before agreeing on a definition, but the decision is probably driven from a data consumer perspective, which demands as much data as possible in a highly structured way resulting in more complex metadata. Data producers, on the other hand, want to reduce their efforts for data collection and might choose a simpler variant. Moreover, top-down standardization is a manual process, which increases costs. In our opinion, top-down MDRs can be advantageous when the scope of an MDR is small, there is high agreement among experts for metadata definitions, or full control of the data is necessary. In contrast to that, the pragmatic bottom-up concept could be of value, when the scope of an MDR is broad and no single ground truth definition exists or is necessary. Due to these different characteristics, bottom-up standardization is unlikely to replace top-down approaches. However, depending on the application it could serve as a useful complement.

### Pragmatic MDR and ISO/IEC 11179 norm for metadata registries

ISO/IEC 11179 specifies a conceptual model for MDRs and metadata representation, which includes a data element definition, conceptual domain, value domain, and data element concept [9]. Such a rigorous data model can improve data definitions, collection guidelines, and quality that ultimately improve the overall data quality of medical data collections [37]. Extensive content curation is necessary to ensure adherence to this data model. In practice, ISO/IEC 11179 compliant MDRs try to fulfill this data model through their top-down approach for standardization. However, an evaluation of caCORE in 2006 identified several limitations concerning inconsistent, insufficient, and redundant content [10]. This evaluation demonstrates the intrinsic difficulties to maintain a consistent and complete ISO/IEC 11179 compliant MDR.

For a pragmatic MDR, ISO/IEC 11179 is not well suited, because deriving the conceptual domain and value domain in an automatic fashion is hard, which would impede automatic data processing. For a pragmatic MDR, it is therefore preferable to use a more relaxed data model to simplify data sharing and obtain a large collection of real-world metadata definitions. Our proof of concept implementation fulfills ISO/IEC 11179 in part, due to the properties of ODM [38]. Using ISO/IEC 11179 for an MDR can be advantageous in similar situations as the top-down standardization approach; when the scope is small and there is high agreement among experts for metadata definitions. However, for the pragmatic MDR concept, the data model is usually too rigorous.

### Limitations

Our definition of a pragmatic MDR along three principles is relatively loose. For example, we do not specify how open access should be ensured or clearly define frequency-based scoring. It is arguable whether our proof of concept implementation satisfies the third principle for open-access. At present, sharing of medical documentation is implemented only through the MDM Portal to exploit its data as initial content and to offer a graphical interface for sharing. Querying and reusing metadata definitions is secured with API-keys. However, necessary keys are provided to all interested parties on request. Moreover, our prototype is limited to ODM metadata; its internal data model and API are derived from this standard. In addition, due to the content of the MDM Portal, we can ensure that our proof of concept is based on real-world metadata definitions and it remains open how to ensure this in other settings.

The proposed bottom-up concept to rank data elements according to their frequency in real-world data collections is certainly a rather simplistic approach to determine their quality and relevance for reuse. Yet, we considered it worthwhile to study its potential to establish an ordering in a large collection of metadata. Also, we performed our quality evaluation of frequent item definitions only with two evaluators with fair agreement and used self-designed questionnaires that were derived from the ODM standard [19]. Our evaluation does not explicitly measure the reliability, validity, and economic factors of a medical item definition. Hence, our evaluation results should be interpreted with caution. We only considered 24 important item concepts from four different categories. The quality of less relevant item definitions might be worse. The same applies to our analysis of cumulative frequencies as well as occurrence ratios and absolute occurrences of the top three search results. While our analysis suggests that certain definitions emerge as de facto standards it is not clear whether this also holds for other item concepts.

Lastly, our overview of existing MDRs and the ISO/IEC 11179 norm is limited. These infrastructures have different guidelines for their content that we subsumed as top-down standardization. This is certainly an oversimplification. We did this to contrast them with our approach for bottom-up standardization.

## Conclusions

In this study, we suggested the pragmatic MDR concept, which enables a community-driven bottom-up approach for standardization of medical metadata. In contrast to existing MDRs, it is based on a large collection of real-world metadata and uses frequency-based scoring of data elements as a proxy for their quality. We successfully implemented a proof of concept application and filled it with 466,569 unique metadata definitions from the MDM Portal. Applications of this prototype in two use cases suggest that it can facilitate the reuse of metadata. Moreover, our analysis for the existence of bottom-up standards showed that for rather simple medical concepts such as laboratory values and vital signs at least weak bottom-up standards emerged in our prototype. For more diverse concepts related to ischaemic heart disease or stroke, such standards could not be determined due to an enormous heterogeneity of data elements. Our evaluation of metadata quality suggests that our proof of concept can offer useful item definitions. In our opinion, a pragmatic MDR is a useful concept alongside existing top-down MDRs that simplifies standardization, gives a broader overview of existing metadata definitions, and offers standards derived from real-world documentation. We think it has potential to facilitate the reuse of data elements during the creation of case report forms and routine documentation.

Future work should consider refined equivalence criteria used for metadata aggregation as we have done it with the data element’s question text. In previous work semantic coding [33, 34, 39], existing common data elements [11], or natural language processing [40] were used to identify equivalent data elements. This would increase the reuse ratio and decrease the long tail of rare metadata definitions. Moreover, it would be of interest to consider a pragmatic MDR that is not restricted to the content of the MDM Portal and allows metadata sharing from different sources. One possibility to realize this is a public API also for sharing and deleting metadata definitions. Most importantly, future work should investigate the utility of the pragmatic MDR concept. For example, by connecting an application to the REST API of our prototype. There are various usage scenarios such as suggestion mechanisms [35] or automatic semantic coding [41] and further experience is necessary to assess the value of this concept.

## Supplementary Information

**Additional file 1.** Questionnaires for the quality evaluation of bottom-up standards.

**Additional file 2.** Study protocol for the quality evaluation of bottom-up standards.

**Additional file 3.** Table with example requests for the REST API of the pragmatic MDR proof of concept.

**Additional file 4.** Overview of item definitions for the quality evaluation of bottom-up standards.

**Additional file 5.** Contingency table and heat map for rater agreement.

**Additional file 6.** Heat map for median ratings for each item concept across both raters and top three search results.

## Data Availability

The datasets used and/or analyzed during the current study are available from the corresponding author on reasonable request.

## Abbreviations

A: Agree
CDASH: Clinical Data Acquisition Standards Harmonization
CDISC: Clinical Data Interchange Standards Consortium
D: Disagree
LOINC: Logical Observation Identifiers Names and Codes
MDM Portal: Portal of Medical Data Models
MDR: Metadata Repository
N: Neither agree nor disagree
Occ: Occurrences
ODM: Operational Data Model
SA: Strongly agree
SD: Strongly disagree
SHIP: Study of Health in Pomerania
UMLS: Unified Medical Language System

## Footnotes

1 We queried the MDM Portal with *"Ischemic heart disease" OR "Coronary heart disease" OR "heart attack"* and *“Stroke”*.

2 Additional file 3 contains more query examples. Further endpoints and parameters are available and documented on the start page of the pragmatic MDR (https://medical-data-models.org/MDR/).
