# Supplementary material for: Pragmatic MDR: a metadata repository with bottom-up standardization of medical metadata through reuse
Source: BMC Med Inform Decis Mak. 2021 May 17;21:160. doi: 10.1186/s12911-021-01524-8 (PMC8130274; doi:10.1186/s12911-021-01524-8)
Supplement: Supplementary file 1 — Additional file 1. Questionnaires for the quality evaluation of bottom-up standards. [file 12911_2021_1524_MOESM1_ESM.pdf]

Item Quality Evaluation

Overview Item Evaluation Criteria

The question text of each item definition should be the main identifier for its semantics. Hence, the question text determines which data an item shall collect. As a consequence, the question cannot be evaluated for its semantic correctness, but only whether it matches formal requirements as clear wording or correct spelling. All other elements of the item definition must be evaluated for their correctness in regard to the item semantics, i.e. if the options offered by the Codelist do not make sense for the item Question, the Codelist should be rated low.

Question: Is the question a suitable label shown to a human user when prompted to provided data for this item on paper or on a screen?

Codelist: Is the discrete set of permitted values with the print /display-forms and actual values sensible for the item definition?

Name: Is the name a proper human readable identifier for the item entity?

DataType: Is the data type general enough to represent the collected data and is it the most restrictive one that still specifies the data correctly?

Length (*required for text/string, optional for integer/float*): Is the length in combination with the data type and range checks sensible for the collected data?

Description: Is the description a meaningful free-text description of the item component?

Alias: Do the alias elements contain a suitable semantic coding for the medical concept of the item?

RangeChecks: Are the range checks in combination with the data type and length sensible for the collected data?

Good match: Is the item definition a reasonable result for the search query?

Relevancy: Is the item definition relevant?

Overview Item data type definitions

<table>
<tr><th>Format Name</th><th>Schema Datatype</th><th>Allowed String Pattern</th></tr>
<tr><td>integer</td><td>xs:integer</td><td>-?digit+</td></tr>
<tr><td>float</td><td>xs:decimal</td><td>-?digit+(.digit+)?</td></tr>
<tr><td>date</td><td>xs:date</td><td>YYYY-MM-DD</td></tr>
<tr><td>time</td><td>xs:time</td><td>hh:mm:ss(.n+)? (((+|-)hh:mm)|Z)?</td></tr>
<tr><td>datetime</td><td>xs:dateTime</td><td>YYYY-MM-DDThh:mm:ss(.n+)?(((+|-)hh:mm)|Z)?</td></tr>
<tr><td>text</td><td>xs:string</td><td>any sequence of characters</td></tr>
<tr><td>string</td><td>xs:string</td><td>Semantically equivalent to text but directly supported as XML Schema datatype</td></tr>
<tr><td>boolean</td><td>xs:boolean</td><td>(true | false | 1 | 0)</td></tr>
<tr><td>double</td><td>xs:string</td><td>(((\+|-)?[0-9]+(\.[0-9]+)?((D|d|E|e)(\+|-)[0-9]+)?)|(-?INF)|(NaN))</td></tr>
<tr><td>hexBinary</td><td>xs:hexBinary</td><td>hex-encoded binary stream data</td></tr>
<tr><td>base64Binary</td><td>xs:base64Binary</td><td>binary stream encoded using Base64 Alphabet</td></tr>
<tr><td>hexFloat</td><td>xs:hexBinary</td><td>up to 16 characters</td></tr>
<tr><td>base64Float</td><td>xs:base64Binary</td><td>up to 12 characters</td></tr>
<tr><td>partialDate</td><td>xs:date</td><td>[YYYY[-MM[-DD]]]</td></tr>
<tr><td>partialTime</td><td>xs:time</td><td>[hh[:mm[:ss(.n+)? (((+|-)hh:mm)|Z)?]]]</td></tr>
<tr><td>partialDatetime</td><td>xs:dateTime</td><td>[YYYY[-MM[-DD[T hh[:mm[:ss(.n+)?((+|-)hh:mm)?]]]]]]</td></tr>
<tr><td>intervalDatetime</td><td>xs:string</td><td>partialDatetime/partialDatetime)|(durationDatetime/partialDatetime)|(partialDatetime/durationDatetime)</td></tr>
<tr><td>durationDatetime</td><td>xs:duration</td><td>(((+|-)?P((((n(n+)?)Y)?((nn+)?)M)?((nn+)?)D)?)(T(((n(n+)?)H)?((n(n+)?)M)?((n(n+)?)((\.n+)?)S)?)?)?|(((n(n+)?)W))))</td></tr>
<tr><td>incompleteDatetime</td><td>xs:string</td><td>[YYYY|-]-[MM |-]-[DD|-]]]T[hh|-]:[mm|-]:[ss.s|-][?(+|-)nn:nn|Z]</td></tr>
<tr><td>incompleteDate</td><td>xs:string</td><td>[YYYY|-]-[MM |-]-[DD|-]</td></tr>
<tr><td>incompleteTime</td><td>xs:string</td><td>T[hh|-]:[mm|-]:[ss.s|-][?(+|-)nn:nn|Z]</td></tr>
<tr><td>URI</td><td>xs:anyURI</td><td></td></tr>
</table>

Item 1/24: “Body Height”

Candidate 1/3

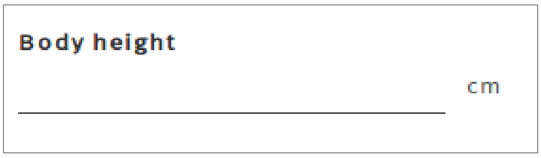

Name: Height
DataType: float
Length: 255
Description:
Alias: Body Height
RangeChecks:

	Strongly disagree (1)	Disagree (2)	Neither agree nor disagree (3)	Agree (4)	Strongly agree (5)
**Question**					
**Codelist**					
**Name**					
**DataType**					
**Length**					
**Description**					
**Alias**					
**RangeChecks**					
**Good match**					
**Relevancy**					

Candidate 2/3

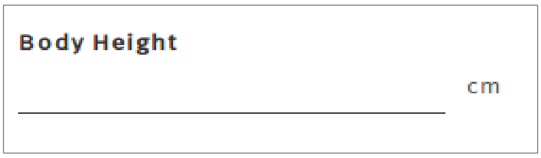

Name: Body Height
DataType: integer
Length: 7
Description:
Alias: Body Height
RangeChecks:

	Strongly disagree (1)	Disagree (2)	Neither agree nor disagree (3)	Agree (4)	Strongly agree (5)
**Question**					
**Codelist**					
**Name**					
**DataType**					
**Length**					
**Description**					
**Alias**					
**RangeChecks**					
**Good match**					
**Relevancy**					

Candidate 3/3

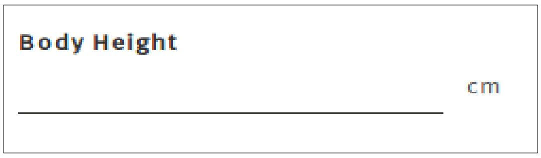

Name: Body Height
DataType: integer
Length:
Description:
Alias: Body Height
RangeChecks:

	Strongly disagree (1)	Disagree (2)	Neither agree nor disagree (3)	Agree (4)	Strongly agree (5)
**Question**					
**Codelist**					
**Name**					
**DataType**					
**Length**					
**Description**					
**Alias**					
**RangeChecks**					
**Good match**					
**Relevancy**					

Item 2/24: “Body Weight”

Candidate 1/3

Body weight

____________________ kg

Name: Weight
DataType: float
Length: 255
Description:
Alias: Body Weight
RangeChecks:

	Strongly disagree (1)	Disagree (2)	Neither agree nor disagree (3)	Agree (4)	Strongly agree (5)
**Question**					
**Codelist**					
**Name**					
**DataType**					
**Length**					
**Description**					
**Alias**					
**RangeChecks**					

**Good match**					
**Relevancy**					

Candidate 2/3

Body weight

____________________ kg

Name: Weight
DataType: float
Length: 3
Description: Body weight
Alias: Body Weight
RangeChecks: >= 0

	Strongly disagree (1)	Disagree (2)	Neither agree nor disagree (3)	Agree (4)	Strongly agree (5)
**Question**					
**Codelist**					
**Name**					
**DataType**					
**Length**					
**Description**					
**Alias**					
**RangeChecks**					

**Good match**					
**Relevancy**					

Candidate 3/3

Body weight

____________________ kg

Name: Body weight
DataType: float
Length: 10
Description:
Alias: Body Weight
RangeChecks:

	Strongly disagree (1)	Disagree (2)	Neither agree nor disagree (3)	Agree (4)	Strongly agree (5)
**Question**					
**Codelist**					
**Name**					
**DataType**					
**Length**					
**Description**					
**Alias**					
**RangeChecks**					

**Good match**					
**Relevancy**					

Item 3/24: “Diastolic BP”

Candidate 1/3

BP:

________________________ mmHg

Name: diastolic blood pressure
DataType: integer
Length: 255
Description: diastolic
Alias: Diastolic blood pressure
RangeChecks:

	Strongly disagree (1)	Disagree (2)	Neither agree nor disagree (3)	Agree (4)	Strongly agree (5)
**Question**					
**Codelist**					
**Name**					
**DataType**					
**Length**					
**Description**					
**Alias**					
**RangeChecks**					

**Good match**					
**Relevancy**					

Candidate 2/3

Semi-supine BP Diastolic

____

Name: diastolic pressure
DataType: text
Length: 255
Description:
Alias: Diastolic blood pressure
RangeChecks:

	Strongly disagree (1)	Disagree (2)	Neither agree nor disagree (3)	Agree (4)	Strongly agree (5)
**Question**					
**Codelist**					
**Name**					
**DataType**					
**Length**					
**Description**					
**Alias**					
**RangeChecks**					

**Good match**					
**Relevancy**					

Candidate 3/3

Standing BP Diastolic

____

Name: diastolic pressure standing
DataType: text
Length: 255
Description:
Alias: Diastolic blood pressure, Standing position
RangeChecks:

	Strongly disagree (1)	Disagree (2)	Neither agree nor disagree (3)	Agree (4)	Strongly agree (5)
**Question**					
**Codelist**					
**Name**					
**DataType**					
**Length**					
**Description**					
**Alias**					
**RangeChecks**					

**Good match**					
**Relevancy**					

Item 4/24: “Systolic BP”

Candidate 1/3

BP:

____________________ mmHg

Name: systolic blood pressure
DataType: integer
Length: 255
Description: systolic
Alias: Systolic Pressure
RangeChecks:

	Strongly disagree (1)	Disagree (2)	Neither agree nor disagree (3)	Agree (4)	Strongly agree (5)
**Question**					
**Codelist**					
**Name**					
**DataType**					
**Length**					
**Description**					
**Alias**					
**RangeChecks**					
**Good match**					
**Relevancy**					

Candidate 2/3

Systolic BP

____________________ mmHg

Name: Systolic Blood Pressure
DataType: integer
Length:
Description: Systolic BP
Alias: Systolic Pressure
RangeChecks:

	Strongly disagree (1)	Disagree (2)	Neither agree nor disagree (3)	Agree (4)	Strongly agree (5)
**Question**					
**Codelist**					
**Name**					
**DataType**					
**Length**					
**Description**					
**Alias**					
**RangeChecks**					
**Good match**					
**Relevancy**					

Candidate 3/3

Semi-supine BP Systolic

____

Name: systolic pressure
DataType: text
Length: 255
Description:
Alias: Systolic Pressure
RangeChecks:

	Strongly disagree (1)	Disagree (2)	Neither agree nor disagree (3)	Agree (4)	Strongly agree (5)
**Question**					
**Codelist**					
**Name**					
**DataType**					
**Length**					
**Description**					
**Alias**					
**RangeChecks**					
**Good match**					
**Relevancy**					

Item 5/24: “Pulse”

Candidate 1/3

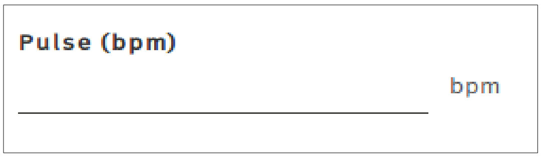

Name: Pulse
DataType: integer
Length: 255
Description:
Alias: Pulse Rate
RangeChecks:

	Strongly disagree (1)	Disagree (2)	Neither agree nor disagree (3)	Agree (4)	Strongly agree (5)
**Question**					
**Codelist**					
**Name**					
**DataType**					
**Length**					
**Description**					
**Alias**					
**RangeChecks**					
**Good match**					
**Relevancy**					

Candidate 2/3

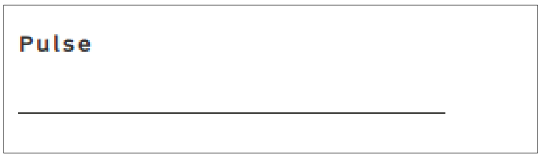

Name: Pulse
DataType: text
Length: 255
Description:
Alias: Pulse Rate
RangeChecks:

	Strongly disagree (1)	Disagree (2)	Neither agree nor disagree (3)	Agree (4)	Strongly agree (5)
**Question**					
**Codelist**					
**Name**					
**DataType**					
**Length**					
**Description**					
**Alias**					
**RangeChecks**					
**Good match**					
**Relevancy**					

Candidate 3/3

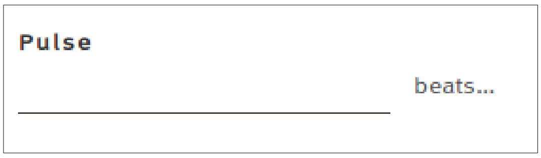

Name: Pulse Rate
DataType: integer
Length: 255
Description: Post-Dose Reading 1: after 10 min in semi-supine position. 2 hours post-dose.
Alias: Pulse Rate
RangeChecks:

	Strongly disagree (1)	Disagree (2)	Neither agree nor disagree (3)	Agree (4)	Strongly agree (5)
**Question**					
**Codelist**					
**Name**					
**DataType**					
**Length**					
**Description**					
**Alias**					
**RangeChecks**					
**Good match**					
**Relevancy**					

Item 6/24: “Body Temperature”

Candidate 1/3

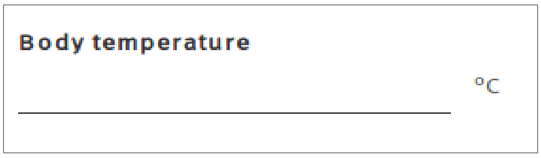

Name: Body temperature
DataType: float
Length: 255
Description: Sub-Itemgroup: Vital Signs
Alias: Body Temperature
RangeChecks:

	Strongly disagree (1)	Disagree (2)	Neither agree nor disagree (3)	Agree (4)	Strongly agree (5)
**Question**					
**Codelist**					
**Name**					
**DataType**					
**Length**					
**Description**					
**Alias**					
**RangeChecks**					
**Good match**					
**Relevancy**					

Candidate 2/3

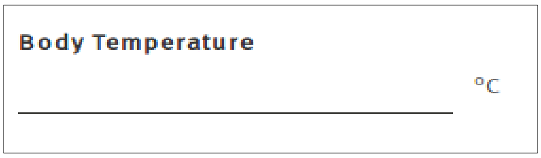

Name: Body Temperature
DataType: float
Length:
Description:
Alias: Body Temperature
RangeChecks:

	Strongly disagree (1)	Disagree (2)	Neither agree nor disagree (3)	Agree (4)	Strongly agree (5)
**Question**					
**Codelist**					
**Name**					
**DataType**					
**Length**					
**Description**					
**Alias**					
**RangeChecks**					
**Good match**					
**Relevancy**					

Candidate 3/3

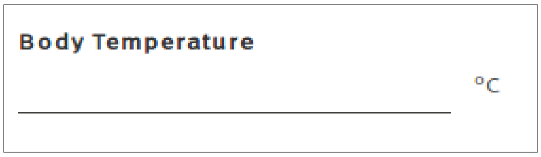

Name: Body Temperature
DataType: float
Length: 255
Description:
Alias: Body Temperature
RangeChecks:

	Strongly disagree (1)	Disagree (2)	Neither agree nor disagree (3)	Agree (4)	Strongly agree (5)
**Question**					
**Codelist**					
**Name**					
**DataType**					
**Length**					
**Description**					
**Alias**					
**RangeChecks**					
**Good match**					
**Relevancy**					

Item 7/24: “Creatinine”

Candidate 1/3

Creatinine

mg/dL

Name: Creatinine, Serum
DataType: float
Length: 5
Description: Creatinine
Alias: Creatinine measurement, serum (procedure)
RangeChecks:

	Strongly disagree (1)	Disagree (2)	Neither agree nor disagree (3)	Agree (4)	Strongly agree (5)
**Question**					
**Codelist**					
**Name**					
**DataType**					
**Length**					
**Description**					
**Alias**					
**RangeChecks**					
**Good match**					
**Relevancy**					

Candidate 2/3

Creatinine

Name: Creatinine
DataType: float
Length: 255
Description:
Alias: Creatinine measurement, serum (procedure)
RangeChecks:

	Strongly disagree (1)	Disagree (2)	Neither agree nor disagree (3)	Agree (4)	Strongly agree (5)
**Question**					
**Codelist**					
**Name**					
**DataType**					
**Length**					
**Description**					
**Alias**					
**RangeChecks**					
**Good match**					
**Relevancy**					

Candidate 3/3

Creatinine Clearance

mL/min

Name: Creatinine Clearance
DataType: float
Length: 7
Description: Creatinine Clearance
Alias: Creatinine clearance measurement
RangeChecks:

	Strongly disagree (1)	Disagree (2)	Neither agree nor disagree (3)	Agree (4)	Strongly agree (5)
**Question**					
**Codelist**					
**Name**					
**DataType**					
**Length**					
**Description**					
**Alias**					
**RangeChecks**					
**Good match**					
**Relevancy**					

Item 8/24: “Hemoglobin”

Candidate 1/3

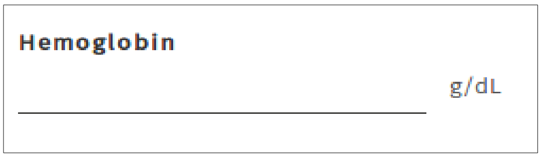

Name: Hemoglobin
DataType: float
Length: 10
Description:
Alias: Hemoglobin
RangeChecks:

	Strongly disagree (1)	Disagree (2)	Neither agree nor disagree (3)	Agree (4)	Strongly agree (5)
**Question**					
**Codelist**					
**Name**					
**DataType**					
**Length**					
**Description**					
**Alias**					
**RangeChecks**					

**Good match**					
**Relevancy**					

Candidate 2/3

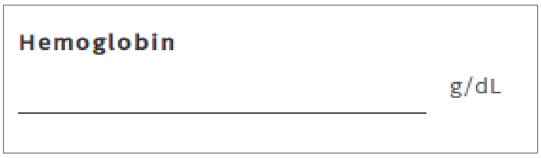

Name: Hemoglobin
DataType: float
Length: 5
Description: Hemoglobin
Alias: Hemoglobin
RangeChecks:

	Strongly disagree (1)	Disagree (2)	Neither agree nor disagree (3)	Agree (4)	Strongly agree (5)
**Question**					
**Codelist**					
**Name**					
**DataType**					
**Length**					
**Description**					
**Alias**					
**RangeChecks**					

**Good match**					
**Relevancy**					

Candidate 3/3

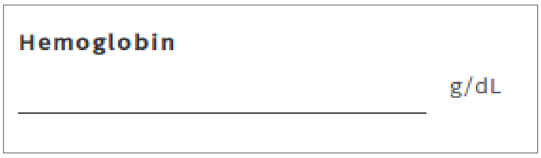

Name: LaboratoryProcedureHemoglobinResultSpecifiedValue
DataType: float
Length: 5
Description:
Alias:
RangeChecks:

	Strongly disagree (1)	Disagree (2)	Neither agree nor disagree (3)	Agree (4)	Strongly agree (5)
**Question**					
**Codelist**					
**Name**					
**DataType**					
**Length**					
**Description**					
**Alias**					
**RangeChecks**					

**Good match**					
**Relevancy**					

Item 9/24: “Potassium”

Candidate 1/3

Potassium

mmol/l

Name: Potassium
DataType: float
Length: 10
Description:
Alias: Potassium measurement
RangeChecks:

	Strongly disagree (1)	Disagree (2)	Neither agree nor disagree (3)	Agree (4)	Strongly agree (5)
**Question**					
**Codelist**					
**Name**					
**DataType**					
**Length**					
**Description**					
**Alias**					
**RangeChecks**					
**Good match**					
**Relevancy**					

Candidate 2/3

Potassium

Name: Potassium
DataType: float
Length: 255
Description:
Alias: Potassium measurement
RangeChecks:

	Strongly disagree (1)	Disagree (2)	Neither agree nor disagree (3)	Agree (4)	Strongly agree (5)
**Question**					
**Codelist**					
**Name**					
**DataType**					
**Length**					
**Description**					
**Alias**					
**RangeChecks**					
**Good match**					
**Relevancy**					

Candidate 3/3

Potassium

mmol/L

Name: Potassium
DataType: float
Length: 255
Description:
Alias: Potassium measurement
RangeChecks:

	Strongly disagree (1)	Disagree (2)	Neither agree nor disagree (3)	Agree (4)	Strongly agree (5)
**Question**					
**Codelist**					
**Name**					
**DataType**					
**Length**					
**Description**					
**Alias**					
**RangeChecks**					
**Good match**					
**Relevancy**					

Item 10/24: “Glucose”

Candidate 1/3

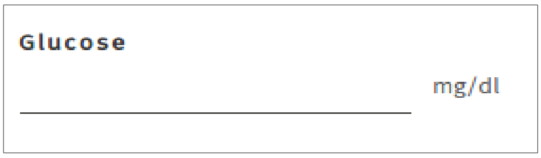

Name: Glucose
DataType: float
Length: 10
Description:
Alias: Plasma Glucose Measurement
RangeChecks:

	Strongly disagree (1)	Disagree (2)	Neither agree nor disagree (3)	Agree (4)	Strongly agree (5)
**Question**					
**Codelist**					
**Name**					
**DataType**					
**Length**					
**Description**					
**Alias**					
**RangeChecks**					

**Good match**					
**Relevancy**					

Candidate 2/3

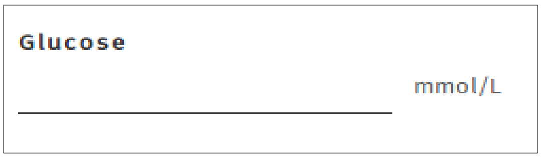

Name: Blood Glucose
DataType: float
Length: 255
Description:
Alias: Plasma Glucose Measurement
RangeChecks:

	Strongly disagree (1)	Disagree (2)	Neither agree nor disagree (3)	Agree (4)	Strongly agree (5)
**Question**					
**Codelist**					
**Name**					
**DataType**					
**Length**					
**Description**					
**Alias**					
**RangeChecks**					

**Good match**					
**Relevancy**					

Candidate 3/3

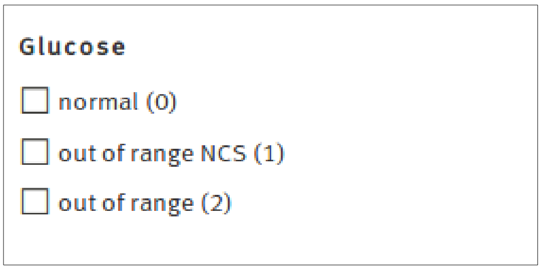

Name: Urine Glucose
DataType: integer
Length: 255
Description:
Alias: Glucose measurement, urine
RangeChecks:

	Strongly disagree (1)	Disagree (2)	Neither agree nor disagree (3)	Agree (4)	Strongly agree (5)
**Question**					
**Codelist**					
**Name**					
**DataType**					
**Length**					
**Description**					
**Alias**					
**RangeChecks**					

**Good match**					
**Relevancy**					

Item 11/24: “Sodium”

Candidate 1/3

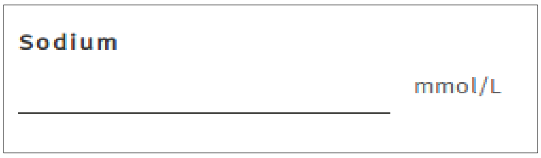

Name: Sodium
DataType: float
Length: 10
Description:
Alias: Sodium measurement
RangeChecks:

	Strongly disagree (1)	Disagree (2)	Neither agree nor disagree (3)	Agree (4)	Strongly agree (5)
**Question**					
**Codelist**					
**Name**					
**DataType**					
**Length**					
**Description**					
**Alias**					
**RangeChecks**					

**Good match**					
**Relevancy**					

Candidate 2/3

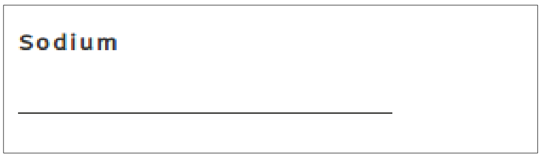

Name: Sodium
DataType: float
Length: 255
Description:
Alias: Sodium measurement
RangeChecks:

	Strongly disagree (1)	Disagree (2)	Neither agree nor disagree (3)	Agree (4)	Strongly agree (5)
**Question**					
**Codelist**					
**Name**					
**DataType**					
**Length**					
**Description**					
**Alias**					
**RangeChecks**					

**Good match**					
**Relevancy**					

Candidate 3/3

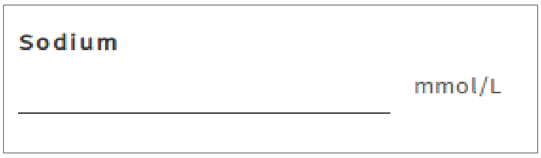

Name: Sodium
DataType: float
Length: 255
Description:
Alias: Sodium measurement
RangeChecks:

	Strongly disagree (1)	Disagree (2)	Neither agree nor disagree (3)	Agree (4)	Strongly agree (5)
**Question**					
**Codelist**					
**Name**					
**DataType**					
**Length**					
**Description**					
**Alias**					
**RangeChecks**					

**Good match**					
**Relevancy**					

Item 12/24: “Urea nitrogen”

Candidate 1/3

Blood urea nitrogen

______________________ mmol/L

Name: Blood Urea Nitrogen
DataType: float
Length: 255
Description:
Alias: Blood urea nitrogen measurement
RangeChecks:

	Strongly disagree (1)	Disagree (2)	Neither agree nor disagree (3)	Agree (4)	Strongly agree (5)
**Question**					
**Codelist**					
**Name**					
**DataType**					
**Length**					
**Description**					
**Alias**					
**RangeChecks**					

**Good match**					
**Relevancy**					

Candidate 2/3

Serum Urea

- ☐ 0-5.9 mmol/L OR 0-.35 g/L (0)
- ☐ 6-9.9 mmol/L OR .36-.59 g/L (1)
- ☐ 10-19.9 mmol/L OR .60-1.19 g/L (3)
- ☐ ≥20 mmol/L OR ≥ 1.20 g/L (5)

Name: Serum Urea
DataType: integer
Length: 255
Description: Serum urea or serum urea nitrogen level
Alias: Urea measurement
RangeChecks:

	Strongly disagree (1)	Disagree (2)	Neither agree nor disagree (3)	Agree (4)	Strongly agree (5)
**Question**					
**Codelist**					
**Name**					
**DataType**					
**Length**					
**Description**					
**Alias**					
**RangeChecks**					

**Good match**					
**Relevancy**					

Candidate 3/3

Serum Urea

- ☐ 0-5.9 mmol/L OR 0-.35 g/L (0)
- ☐ 6-9.9 mmol/L OR .36-.59 g/L (1)
- ☐ 10-19.9 mmol/L OR .60-1.19 g/L (3)
- ☐ ≥20 mmol/L OR ≥ 1.20 g/L (5)

Name: Serum Urea Nitrogen
DataType: integer
Length: 255
Description: Serum urea or serum urea nitrogen level
Alias: Blood urea nitrogen measurement
RangeChecks:

	Strongly disagree (1)	Disagree (2)	Neither agree nor disagree (3)	Agree (4)	Strongly agree (5)
**Question**					
**Codelist**					
**Name**					
**DataType**					
**Length**					
**Description**					
**Alias**					
**RangeChecks**					

**Good match**					
**Relevancy**					

Item 13/24: “Myocardial Infarction”

Candidate 1/3

Myocardial infarction

☐ Yes

☐ No

Name: myocardial infarction
DataType: boolean
Length:
Description: Myocardial infarction
Alias: Myocardial Infarction
RangeChecks:

	Strongly disagree (1)	Disagree (2)	Neither agree nor disagree (3)	Agree (4)	Strongly agree (5)
**Question**					
**Codelist**					
**Name**					
**DataType**					
**Length**					
**Description**					
**Alias**					
**RangeChecks**					
**Good match**					
**Relevancy**					

Candidate 2/3

Myocardial Infarction

☐ no (0)

☐ yes (1)

Name: Myocardial Infarction
DataType: integer
Length: 255
Description:
Alias: Myocardial Infarction
RangeChecks:

	Strongly disagree (1)	Disagree (2)	Neither agree nor disagree (3)	Agree (4)	Strongly agree (5)
**Question**					
**Codelist**					
**Name**					
**DataType**					
**Length**					
**Description**					
**Alias**					
**RangeChecks**					
**Good match**					
**Relevancy**					

Candidate 3/3

Myocardial infarction

☐ Yes

☐ No

Name: Myocardial infarction
DataType: boolean
Length:
Description:
Alias: Myocardial Infarction
RangeChecks:

	Strongly disagree (1)	Disagree (2)	Neither agree nor disagree (3)	Agree (4)	Strongly agree (5)
**Question**					
**Codelist**					
**Name**					
**DataType**					
**Length**					
**Description**					
**Alias**					
**RangeChecks**					
**Good match**					
**Relevancy**					

Item 14/24: “Coronary Artery Bypass Surgery”

Candidate 1/3

CABG

☐ Yes

☐ No

Name: History of coronary artery bypass grafting
DataType: boolean
Length:
Description: Coronary Artery Bypass Surgery
Alias: History of coronary artery bypass grafting
RangeChecks:

	Strongly disagree (1)	Disagree (2)	Neither agree nor disagree (3)	Agree (4)	Strongly agree (5)
**Question**					
**Codelist**					
**Name**					
**DataType**					
**Length**					
**Description**					
**Alias**					
**RangeChecks**					
**Good match**					
**Relevancy**					

Candidate 2/3

Coronary artery bypass surgery (CABG-Op)

☐ Yes

☐ No

Name: CABG-Op
DataType: boolean
Length:
Description: Coronary artery bypass surgery (CABG-Op)
Alias: Coronary Artery Bypass Surgery
RangeChecks:

	Strongly disagree (1)	Disagree (2)	Neither agree nor disagree (3)	Agree (4)	Strongly agree (5)
**Question**					
**Codelist**					
**Name**					
**DataType**					
**Length**					
**Description**					
**Alias**					
**RangeChecks**					
**Good match**					
**Relevancy**					

Candidate 3/3

Coronary Artery Bypass Surgery

☐ Yes

☐ No

Name: Coronary Artery Bypass Surgery
DataType: boolean
Length:
Description:
Alias: Coronary Artery Bypass Surgery
RangeChecks:

	Strongly disagree (1)	Disagree (2)	Neither agree nor disagree (3)	Agree (4)	Strongly agree (5)
**Question**					
**Codelist**					
**Name**					
**DataType**					
**Length**					
**Description**					
**Alias**					
**RangeChecks**					
**Good match**					
**Relevancy**					

Item 15/24: “Angina Pectoris”

Candidate 1/3

Angina pectoris

☐ I. Ordinary physical activity does not cause angina (I. Ordinary physical activity does not cause angina)

☐ II. Slight limitation of ordinary activity (II. Slight limitation of ordinary activity)

☐ III. Marked limitation of ordinary activity (III. Marked limitation of ordinary activity)

☐ IV. Inability to carry out any physical activity without discomfort - angina syndrome may be present at rest (IV. Inability to carry out any physical activity without discomfort - angina syndrome may be present at rest)

Name: Angina pectoris
DataType: text
Length:
Description:
Alias: Angina Pectoris
RangeChecks:

	Strongly disagree (1)	Disagree (2)	Neither agree nor disagree (3)	Agree (4)	Strongly agree (5)
**Question**					
**Codelist**					
**Name**					
**DataType**					
**Length**					
**Description**					
**Alias**					
**RangeChecks**					
**Good match**					
**Relevancy**					

Candidate 2/3

Angina

☐ Yes

☐ No

Name: History of Angina pectoris
DataType: boolean
Length:
Description: Angina pectoris
Alias: H/O: angina pectoris
RangeChecks:

	Strongly disagree (1)	Disagree (2)	Neither agree nor disagree (3)	Agree (4)	Strongly agree (5)
**Question**					
**Codelist**					
**Name**					
**DataType**					
**Length**					
**Description**					
**Alias**					
**RangeChecks**					
**Good match**					
**Relevancy**					

Candidate 3/3

Angina Pectoris

- ☐ yes (1)
- ☐ no (2)
- ☐ unknown (3)

Name: angina
DataType: text
Length: 80
Description:
Alias: Angina Pectoris
RangeChecks:

	Strongly disagree (1)	Disagree (2)	Neither agree nor disagree (3)	Agree (4)	Strongly agree (5)
**Question**					
**Codelist**					
**Name**					
**DataType**					
**Length**					
**Description**					
**Alias**					
**RangeChecks**					

**Good match**					
**Relevancy**					

Item 16/24: “Myocardial Ischemia”

Candidate 1/3

evidence of myocardial ischemia

☐ Yes

☐ No

Name: Myocardial Ischemia Evidence of
DataType: boolean
Length:
Description:
Alias: Myocardial Ischemia, Evidence of (contextual qualifier)
RangeChecks:

	Strongly disagree (1)	Disagree (2)	Neither agree nor disagree (3)	Agree (4)	Strongly agree (5)
**Question**					
**Codelist**					
**Name**					
**DataType**					
**Length**					
**Description**					
**Alias**					
**RangeChecks**					
**Good match**					
**Relevancy**					

Candidate 2/3

exercise-induced myocardial ischemia

☐ Yes

☐ No

Name: Myocardial Ischemia exercise induced
DataType: boolean
Length:
Description:
Alias: Myocardial Ischemia, exercise induced
RangeChecks:

	Strongly disagree (1)	Disagree (2)	Neither agree nor disagree (3)	Agree (4)	Strongly agree (5)
**Question**					
**Codelist**					
**Name**					
**DataType**					
**Length**					
**Description**					
**Alias**					
**RangeChecks**					
**Good match**					
**Relevancy**					

Candidate 3/3

5. Other interventions: [6] ECG

☐ Yes

☐ No

Name: Other interventions ECG
DataType: boolean
Length:
Description: Select all that apply. Complete the ECG evidence of myocardial ischemia/infarction form for ALL ECGs related to this event.
Alias: Electrocardiogram, Other
RangeChecks:

	Strongly disagree (1)	Disagree (2)	Neither agree nor disagree (3)	Agree (4)	Strongly agree (5)
**Question**					
**Codelist**					
**Name**					
**DataType**					
**Length**					
**Description**					
**Alias**					
**RangeChecks**					
**Good match**					
**Relevancy**					

Item 17/24: “Coronary heart disease”

Candidate 1/3

Any contraindication to the use of Adrenaline

☐ Yes

☐ No

Name: Contraindication
DataType: boolean
Length:
Description: i.e: severe acute coronary heart disease, severe hypertension
Alias: Medical contraindication
RangeChecks:

	Strongly disagree (1)	Disagree (2)	Neither agree nor disagree (3)	Agree (4)	Strongly agree (5)
**Question**					
**Codelist**					
**Name**					
**DataType**					
**Length**					
**Description**					
**Alias**					
**RangeChecks**					
**Good match**					
**Relevancy**					

Candidate 2/3

Coronary Artery Disease (heart disease)

☐ No (No)

☐ Yes (Yes)

☐ Unknown (Unknown)

Name: ChronicDiseaseCoronaryArteryInd-3
DataType: text
Length: 7
Description:
Alias:
RangeChecks:

	Strongly disagree (1)	Disagree (2)	Neither agree nor disagree (3)	Agree (4)	Strongly agree (5)
**Question**					
**Codelist**					
**Name**					
**DataType**					
**Length**					
**Description**					
**Alias**					
**RangeChecks**					
**Good match**					
**Relevancy**					

Candidate 3/3

coronary heart disease

☐ Yes

☐ No

Name: Coronary heart disease
DataType: boolean
Length:
Description:
Alias: Coronary heart disease
RangeChecks:

	Strongly disagree (1)	Disagree (2)	Neither agree nor disagree (3)	Agree (4)	Strongly agree (5)
**Question**					
**Codelist**					
**Name**					
**DataType**					
**Length**					
**Description**					
**Alias**					
**RangeChecks**					
**Good match**					
**Relevancy**					

Item 18/24: “Coronary revascularisation”

Candidate 1/3

[4] Non-coronary revascularisation

☐ Yes

☐ No

Name: Hospitalisation for Non-coronary revascularisation
DataType: boolean
Length:
Description:
Alias: Coronary revascularisation
RangeChecks:

	Strongly disagree (1)	Disagree (2)	Neither agree nor disagree (3)	Agree (4)	Strongly agree (5)
**Question**					
**Codelist**					
**Name**					
**DataType**					
**Length**					
**Description**					
**Alias**					
**RangeChecks**					
**Good match**					
**Relevancy**					

Candidate 2/3

4. Has the subject undergone a coronary revascularisation since the last visit?

☐ Yes (Y)

☐ No (N)

☐ Question not asked (Z)

Name: coronary revascularisation
DataType: text
Length: 255
Description: Yes - complete SAE and Revascularisation endpoint form
Alias: Coronary revascularisation, Percutaneous Coronary Intervention
RangeChecks:

	Strongly disagree (1)	Disagree (2)	Neither agree nor disagree (3)	Agree (4)	Strongly agree (5)
**Question**					
**Codelist**					
**Name**					
**DataType**					
**Length**					
**Description**					
**Alias**					
**RangeChecks**					
**Good match**					
**Relevancy**					

Candidate 3/3

Time of revascularisation percutaneous coronary intervention

____

	Strongly disagree (1)	Disagree (2)	Neither agree nor disagree (3)	Agree (4)	Strongly agree (5)
**Question**					
**Codelist**					
**Name**					
**DataType**					
**Length**					
**Description**					
**Alias**					
**RangeChecks**					
**Good match**					
**Relevancy**					

Name: Revascularisation percutaneous coronary intervention date
DataType: date
Length:
Description:
Person—revascularisation percutaneous coronary intervention date, DDMMYYYY

Obligation: Conditional

Identifying and definitional attributes
Short name: Date of revascularisation percutaneous coronary intervention
Synonymous names: Revascularisation PCI date
METeOR identifier: 359731
Registration status: Health, Standard 01/10/2008
Definition: The date when a percutaneous coronary intervention (PCI) is performed for revascularisation.
Data Element Concept: Person—revascularisation percutaneous coronary intervention date
Value domain attributes
Representational attributes
Representation class: Date
Data type: Date/Time
Format: DDMMYYYY
Maximum character length: 8
Data set specification specific attributes
Coronary artery cluster

Conditional obligation: Record when a percutaneous coronary intervention is performed for revascularisation.
Data element attributes
Collection and usage attributes
Guide for use: Revascularisation PCI relates to balloon angioplasty inflation and/or stent implantation performed for subsequent restoration of blood flow.
Comments: Routine revascularisation PCI may be performed after ST-segment-elevation myocardial infarction for people with objective evidence of recurrent myocardial infarction in whom there is spontaneous or inducible ischaemia or haemodynamic instability. Revascularisation PCI may also be performed for treatment of high-risk non-ST-segment-elevation acute coronary syndrome.
Source and reference attributes
Reference documents: National Heart Foundation of Australia & Cardiac Society of Australia and New Zealand. Guidelines for the management of acute coronary syndromes 2006. Med J Aust 2006; 184; S1-S32. © MJA 2006
Relational attributes
Implementation in Data Set Specifications: Coronary artery cluster Health, Standard 01/10/2008
Alias:
RangeChecks:

Item 19/24: “Cerebrovascular accident”

Candidate 1/3

Cerebrovascular Accident

☐ no (0)

☐ yes (1)

Name: Cerebrovascular Accident
DataType: integer
Length: 255
Description:
Alias: Cerebrovascular accident
RangeChecks:

	Strongly disagree (1)	Disagree (2)	Neither agree nor disagree (3)	Agree (4)	Strongly agree (5)
**Question**					
**Codelist**					
**Name**					
**DataType**					
**Length**					
**Description**					
**Alias**					
**RangeChecks**					
**Good match**					
**Relevancy**					

Candidate 2/3

Cerebrovascular accident within one yar

☐ Yes

☐ No

Name: Stroke
DataType: boolean
Length:
Description: Cerebrovascular accident within one yar
Alias: Cerebrovascular accident
RangeChecks:

	Strongly disagree (1)	Disagree (2)	Neither agree nor disagree (3)	Agree (4)	Strongly agree (5)
**Question**					
**Codelist**					
**Name**					
**DataType**					
**Length**					
**Description**					
**Alias**					
**RangeChecks**					
**Good match**					
**Relevancy**					

Candidate 3/3

Cerebrovascular disease

☐ No (1)

☐ Yes (2)

☐ Not evaluated (3)

Name: Cerebrovascular disease
DataType: integer
Length: 255
Description: Transient ischemic attack or cerebrovascular accident
Alias: Cerebrovascular Disorders
RangeChecks:

	Strongly disagree (1)	Disagree (2)	Neither agree nor disagree (3)	Agree (4)	Strongly agree (5)
**Question**					
**Codelist**					
**Name**					
**DataType**					
**Length**					
**Description**					
**Alias**					
**RangeChecks**					
**Good match**					
**Relevancy**					

Item 20/24: “Hemorrhage”

Candidate 1/3

Is a vitreous hemorrhage present?

- ☐ No (1)
- ☐ Trace (2)
- ☐ 1+ (3)
- ☐ 2+ (4)
- ☐ 3+ (5)
- ☐ 4+ (6)
- ☐ Note Done (7)

Name: Vitreous Hemorrhage
DataType: integer
Length: 255
Description:
Alias: Vitreous Hemorrhage
RangeChecks:

	Strongly disagree (1)	Disagree (2)	Neither agree nor disagree (3)	Agree (4)	Strongly agree (5)
**Question**					
**Codelist**					
**Name**					
**DataType**					
**Length**					
**Description**					
**Alias**					
**RangeChecks**					
**Good match**					
**Relevancy**					

Candidate 2/3

Hemorrhage

- ☐ Yes
- ☐ No

Name: Hemorrhage
DataType: boolean
Length:
Description:
Alias:
RangeChecks:

	Strongly disagree (1)	Disagree (2)	Neither agree nor disagree (3)	Agree (4)	Strongly agree (5)
**Question**					
**Codelist**					
**Name**					
**DataType**					
**Length**					
**Description**					
**Alias**					
**RangeChecks**					
**Good match**					
**Relevancy**					

Candidate 3/3

Retinal Hemorrhage

- ☐ Yes
- ☐ No

Name: Retinal Hemorrhage
DataType: boolean
Length:
Description:
Alias: Retinal Hemorrhage
RangeChecks:

	Strongly disagree (1)	Disagree (2)	Neither agree nor disagree (3)	Agree (4)	Strongly agree (5)
**Question**					
**Codelist**					
**Name**					
**DataType**					
**Length**					
**Description**					
**Alias**					
**RangeChecks**					
**Good match**					
**Relevancy**					

Item 21/24: “Transient Ischemic Attack”

Candidate 1/3

History of transient ischemic attack (TIA)

☐ Yes

☐ No

Name: History of TIA
DataType: boolean
Length:
Description: History of transient ischemic attack (TIA)
Alias: H/O: TIA
RangeChecks:

	Strongly disagree (1)	Disagree (2)	Neither agree nor disagree (3)	Agree (4)	Strongly agree (5)
**Question**					
**Codelist**					
**Name**					
**DataType**					
**Length**					
**Description**					
**Alias**					
**RangeChecks**					
**Good match**					
**Relevancy**					

Candidate 2/3

Transient Ischemic Attack

☐ no (0)

☐ yes (1)

Name: Transient Ischemic Attack
DataType: integer
Length: 255
Description:
Alias: Transient Ischemic Attack
RangeChecks:

	Strongly disagree (1)	Disagree (2)	Neither agree nor disagree (3)	Agree (4)	Strongly agree (5)
**Question**					
**Codelist**					
**Name**					
**DataType**					
**Length**					
**Description**					
**Alias**					
**RangeChecks**					
**Good match**					
**Relevancy**					

Candidate 3/3

Stroke/transient ischemic attack (TIA)

☐ Yes

☐ No

Name: Stroke or TIA
DataType: boolean
Length: 7
Description: Stroke/transient ischemic attack (TIA)
Alias: Yes, No, or Unknown Response
RangeChecks:

	Strongly disagree (1)	Disagree (2)	Neither agree nor disagree (3)	Agree (4)	Strongly agree (5)
**Question**					
**Codelist**					
**Name**					
**DataType**					
**Length**					
**Description**					
**Alias**					
**RangeChecks**					
**Good match**					
**Relevancy**					

Item 22/24: “Muscle Weakness”

Candidate 1/3

Muscle weakness

☐ Yes

☐ No

Name: Muscle weakness
DataType: boolean
Length:
Description:
Alias: Muscle Weakness
RangeChecks:

	Strongly disagree (1)	Disagree (2)	Neither agree nor disagree (3)	Agree (4)	Strongly agree (5)
**Question**					
**Codelist**					
**Name**					
**DataType**					
**Length**					
**Description**					
**Alias**					
**RangeChecks**					
**Good match**					
**Relevancy**					

Candidate 2/3

39. Muscle weakness

☐ Not limited ([C08])

☐ Limited a little ([C09])

☐ Limited a lot ([C10])

Name: Muscle weakness
DataType: text
Length: 255
Description:
Alias: Muscle Weakness
RangeChecks:

	Strongly disagree (1)	Disagree (2)	Neither agree nor disagree (3)	Agree (4)	Strongly agree (5)
**Question**					
**Codelist**					
**Name**					
**DataType**					
**Length**					
**Description**					
**Alias**					
**RangeChecks**					
**Good match**					
**Relevancy**					

Candidate 3/3

Musculoskeletal Muscle atrophy or weakness

☐ Yes

☐ No

Name: muscle atrophy or weakness
DataType: boolean
Length:
Description:
Alias: Muscular Atrophy, Muscle Weakness
RangeChecks:

	Strongly disagree (1)	Disagree (2)	Neither agree nor disagree (3)	Agree (4)	Strongly agree (5)
**Question**					
**Codelist**					
**Name**					
**DataType**					
**Length**					
**Description**					
**Alias**					
**RangeChecks**					
**Good match**					
**Relevancy**					

Item 23/24: “grip strength test left hand”

Candidate 1/3

Left Grip Strength Max-Grip Test 1

____

Name: Left Grip Strength
DataType: integer
Length: 7
Description:
Alias: grip strength test left hand
RangeChecks:

	Strongly disagree (1)	Disagree (2)	Neither agree nor disagree (3)	Agree (4)	Strongly agree (5)
**Question**					
**Codelist**					
**Name**					
**DataType**					
**Length**					
**Description**					
**Alias**					
**RangeChecks**					

**Good match**					
**Relevancy**					

Candidate 2/3

Left Grip Strength Max-Grip Test 1

☐ pounds (lb)

☐ kilograms (kg)

☐ newtons (N)

Name: Left Grip Strength
DataType: integer
Length: 7
Description:
Alias: grip strength test left hand
RangeChecks:

	Strongly disagree (1)	Disagree (2)	Neither agree nor disagree (3)	Agree (4)	Strongly agree (5)
**Question**					
**Codelist**					
**Name**					
**DataType**					
**Length**					
**Description**					
**Alias**					
**RangeChecks**					

**Good match**					
**Relevancy**					

Candidate 3/3

Left Grip Strength Max-Grip Test 2

____

Name: Left Grip Strength
DataType: integer
Length: 7
Description:
Alias: grip strength test left hand
RangeChecks:

	Strongly disagree (1)	Disagree (2)	Neither agree nor disagree (3)	Agree (4)	Strongly agree (5)
**Question**					
**Codelist**					
**Name**					
**DataType**					
**Length**					
**Description**					
**Alias**					
**RangeChecks**					

**Good match**					
**Relevancy**					

Item 24/24: “Dysarthria”

Candidate 1/3

Dysarthria

☐ yes (1)

Name: Dysarthrie
DataType: integer
Length:
Description: Dysarthrie
Alias: Dysarthria
RangeChecks:

	Strongly disagree (1)	Disagree (2)	Neither agree nor disagree (3)	Agree (4)	Strongly agree (5)
**Question**					
**Codelist**					
**Name**					
**DataType**					
**Length**					
**Description**					
**Alias**					
**RangeChecks**					

**Good match**					
**Relevancy**					

Candidate 2/3

Dysarthria

☐ Yes (1)

☐ No (2)

☐ Unsure (3)

☐ Not recorded (4)

Name: Neurological impairment: Dysarthria
DataType: integer
Length: 255
Description:
Alias: Neurologic Deficits; Dysarthria
RangeChecks:

	Strongly disagree (1)	Disagree (2)	Neither agree nor disagree (3)	Agree (4)	Strongly agree (5)
**Question**					
**Codelist**					
**Name**					
**DataType**					
**Length**					
**Description**					
**Alias**					
**RangeChecks**					

**Good match**					
**Relevancy**					

Candidate 3/3

Dysarthria

☐ Yes

☐ No

Name: Dysarthria
DataType: boolean
Length:
Description:
Alias: Dysarthria:Find:Pt:^Patient:Ord:NIHSS
RangeChecks:

	Strongly disagree (1)	Disagree (2)	Neither agree nor disagree (3)	Agree (4)	Strongly agree (5)
**Question**					
**Codelist**					
**Name**					
**DataType**					
**Length**					
**Description**					
**Alias**					
**RangeChecks**					

**Good match**					
**Relevancy**					
