# Supplementary material for: Pragmatic MDR: a metadata repository with bottom-up standardization of medical metadata through reuse
Source: BMC Med Inform Decis Mak. 2021 May 17;21:160. doi: 10.1186/s12911-021-01524-8 (PMC8130274; doi:10.1186/s12911-021-01524-8)
Supplement: Supplementary file 2 — Additional file 2. Study protocol for the quality evaluation of bottom-up standards. [file 12911_2021_1524_MOESM2_ESM.pdf]

Study Protocol for Conceptual Evaluation of Pragmatic MDR

This study protocol aims to describe the analysis methods for the conceptual evaluation of the pragmatic metadata repository (MDR). We want to define these methods a priori to prevent any bias that might be introduced after observing the collected data. Alongside this protocol, we provide an R script for analysis that contains the described methods. We use random data from five normal distributions to test the script and to provide example outputs for this protocol. For the final analysis, the observed data will be used instead. In case the collected data leads to a situation that forces us to modify the described methods or to add additional methods, we will describe and justify those in the final report. Stefan Hegselmann and Michael Storck verify this protocol and control the adherence.

Brief Evaluation Description

For the conceptual evaluation, we want to verify whether frequent item definitions are of high quality. To this end, we query the pragmatic MDR with 24 common item names that we chose from four groups: six CDASH vital signs, six most frequent LOINC codes regarding their “rank”, six most frequent *ischaemic heart disease* related items in the MDM-Portal, and six most frequent *stroke* related items in the MDM-Portal. In the first two cases, we use the item names, e.g. “Body Height” or “Creatinine”, to query the pragmatic MDR prototype. In the latter two cases, we identify the most frequent Unified Medical Language System (UMLS) semantic concepts in the MDM-Portal associated with the diseases and use the concept names to query the MDR. For each one of the 24 queries we choose the top three hits in the pragmatic MDR, because these are the most frequent and best matching item definitions for this query. Hence, in total 72 item definitions are evaluated. Two medical data managers perform the evaluation separately regarding the following eight categories: Question, CodeList, Name, DataType, Length, Description, Alias, RangeCheck. Furthermore, the evaluators should decide whether the identified candidate is a good match for the query and whether it is relevant for being used in a study documentation. Rating is done with an ordinal Likert Scale from one to five.

Descriptive Statistics for Categories and Raters

To obtain an overview of the ratings for each category (Question, CodeList…) and to assess the overall scores of each rater, we present the scores assigned to each category by each rater as shown in Table 1. The sum of all scores for each rater are presented as well to give an impression of the overall rating behavior. Moreover, the median rating is highlighted in bold to show a possible tendency of the data. An additional column for not applicable shows the number of categories that could not be rated, for example, because a category such as “Length” is not defined. Note that not applicable always applies to both raters. Hence, the sum of all column values for one rater in addition to the not applicable items always yields as total of 72 rated item definitions.

Table 1. Example table for descriptive statistics for categories and raters with randomly generated data.

		**Rater A**					**Rater B**				
	**NA**	**SD**	**D**	**N**	**A**	**SA**	**SD**	**D**	**N**	**A**	**SA**
Question	8	**32**	**19**	8	5	0	**36**	20	6	2	8
CodeList	8	**40**	15	7	2	0	**50**	5	7	2	0
Name	7	19	**21**	12	7	6	27	**23**	9	4	2
DataType	7	28	**15**	11	10	1	25	**18**	12	5	5
Length	8	11	6	**20**	18	9	15	10	**12**	12	15
Description	8	10	16	**13**	11	14	12	15	**13**	13	11
Alias	7	3	5	13	**21**	23	3	5	11	**16**	30
RangeCheck	8	0	7	18	**21**	18	3	7	11	**23**	20
Good match	8	0	2	4	23	**35**	0	1	8	14	**41**
Relevancy	8	0	4	5	9	**46**	0	4	4	16	**40**
Total	77	143	110	**111**	127	152	171	108	**93**	107	164

Descriptive Statistics for Item Concepts

We also want to assess the item quality for each of the 24 item concepts more specifically. To this end, we take the three top items we identified for each item concept and take the scores of both raters for each category of these items. We take the median value of these scores and visualize it with a blue to white to red color map as shown in Table 2. We have to take the median of an even number of scores, which is not defined for ordinal values. However, to visualize the situation that the two center values are not equal, we use a color in between these two values. Even though this can be considered as treating the ordinal data as interval data, we think it is the best compromise to deal with this situation and visualize the difference.

Table 2. Example table for descriptive statistics for item concepts with randomly generated data.

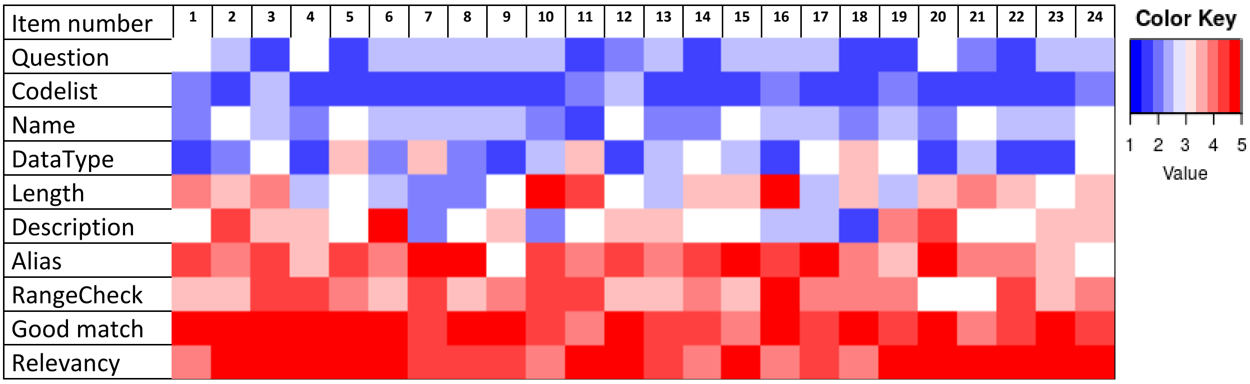

Interrater Reliability

To get an impression of the interrater reliability, we will present a contingency table for both raters and their associated Likert Scale ratings. Furthermore, we will visualize this table as a heatmap in the default coloring of R. These two figures might be placed in the appendix depending on their relevance for the manuscript. Moreover, we will calculate and report Krippendorff's alpha coefficient with bootstrap confidence intervals as a statistical measure for interrater agreement (in the example R script with randomly generated data: K_{alpha} = 0.49, 95% CI 0.42-0.54) [1].

Table 3. Example contingency table with randomly generated data.

		Rater A				
		SD	D	N	A	SD
Rater B	SD	72	36	21	6	8
	D	41	20	18	14	17
	N	28	25	14	21	23
	A	22	14	15	27	49
	SA	8	13	25	39	67

Figure 1. Example heatmap for contingency table with randomly generated data

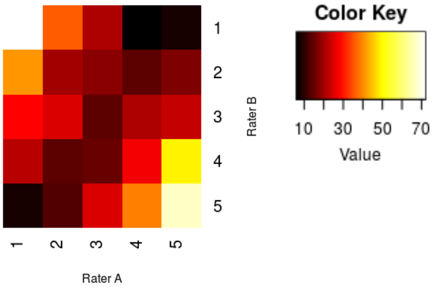

References

1. Zapf A, Castell S, Morawietz L, Karch A. Measuring inter-rater reliability for nominal data – which coefficients and confidence intervals are appropriate? BMC Medical Research Methodology. 2016;16:93. doi:10.1186/s12874-016-0200-9.

R Script

```
# Data analysis for pragamtic MDR paper
library(data.table)
library(dplyr)
library(plyr)
library(gplots)
library(lpSolve)
library(irr)

# Import function for Krippendorff's alpha from paper
# "Measuring inter-rater reliability for nominal data – which coefficients and confidence intervals are appropriate?"
source(k_alpha.R)

likert_ordinals <- factor(1:5, labels=c("SD", "D", "N", "A", "SA"), ordered=TRUE)

# Simulation with random data
set.seed(42)

## For each evaluator there are 72 items to assess with eight categories each
question_a <- sample(likert_ordinals, 72, replace=TRUE, prob=c(0.6, 0.25, 0.1, 0.05, 0.0))
question_b <- sample(likert_ordinals, 72, replace=TRUE, prob=c(0.6, 0.25, 0.1, 0.05, 0.0))
empty_question <- as.integer(runif(8, min=0, max=72))
question_a[empty_question] <- NA
question_b[empty_question] <- NA

codelist_a <- sample(likert_ordinals, 72, replace=TRUE, prob=c(0.6, 0.25, 0.1, 0.05, 0.0))
codelist_b <- sample(likert_ordinals, 72, replace=TRUE, prob=c(0.6, 0.25, 0.1, 0.05, 0.0))
empty_codelist <- as.integer(runif(8, min=0, max=72))
codelist_a[empty_codelist] <- NA
codelist_b[empty_codelist] <- NA

name_a <- sample(likert_ordinals, 72, replace=TRUE, prob=c(0.35, 0.3, 0.2, 0.1, 0.05))
name_b <- sample(likert_ordinals, 72, replace=TRUE, prob=c(0.35, 0.3, 0.2, 0.1, 0.05))
empty_name <- as.integer(runif(8, min=0, max=72))
name_a[empty_name] <- NA
name_b[empty_name] <- NA

datatype_a <- sample(likert_ordinals, 72, replace=TRUE, prob=c(0.35, 0.3, 0.2, 0.1, 0.05))
datatype_b <- sample(likert_ordinals, 72, replace=TRUE, prob=c(0.35, 0.3, 0.2, 0.1, 0.05))
empty_datatype <- as.integer(runif(8, min=0, max=72))
datatype_a[empty_datatype] <- NA
datatype_b[empty_datatype] <- NA

length_a <- sample(likert_ordinals, 72, replace=TRUE, prob=c(0.2, 0.2, 0.2, 0.2, 0.2))
length_b <- sample(likert_ordinals, 72, replace=TRUE, prob=c(0.2, 0.2, 0.2, 0.2, 0.2))
empty_length <- as.integer(runif(8, min=0, max=72))
length_a[empty_length] <- NA
length_b[empty_length] <- NA

description_a <- sample(likert_ordinals, 72, replace=TRUE, prob=c(0.2, 0.2, 0.2, 0.2, 0.2))
description_b <- sample(likert_ordinals, 72, replace=TRUE, prob=c(0.2, 0.2, 0.2, 0.2, 0.2))
empty_description <- as.integer(runif(8, min=0, max=72))
description_a[empty_description] <- NA
description_b[empty_description] <- NA

alias_a <- sample(likert_ordinals, 72, replace=TRUE, prob=c(0.05, 0.1, 0.2, 0.3, 0.35))
alias_b <- sample(likert_ordinals, 72, replace=TRUE, prob=c(0.05, 0.1, 0.2, 0.3, 0.35))
empty_alias <- as.integer(runif(8, min=0, max=72))
alias_a[empty_alias] <- NA
alias_b[empty_alias] <- NA

rangechecks_a <- sample(likert_ordinals, 72, replace=TRUE, prob=c(0.05, 0.1, 0.2, 0.3, 0.35))
rangechecks_b <- sample(likert_ordinals, 72, replace=TRUE, prob=c(0.05, 0.1, 0.2, 0.3, 0.35))
empty_rangechecks <- as.integer(runif(8, min=0, max=72))
rangechecks_a[empty_rangechecks] <- NA
rangechecks_b[empty_rangechecks] <- NA

goodmatch_a <- sample(likert_ordinals, 72, replace=TRUE, prob=c(0.0, 0.05, 0.1, 0.25, 0.6))
goodmatch_b <- sample(likert_ordinals, 72, replace=TRUE, prob=c(0.0, 0.05, 0.1, 0.25, 0.6))
empty_goodmatch <- as.integer(runif(8, min=0, max=72))
goodmatch_a[empty_goodmatch] <- NA
```

```
goodmatch_b[empty_goodmatch] <- NA

relevancy_a <- sample(likert_ordinals, 72, replace=TRUE, prob=c(0.0, 0.05, 0.1, 0.25, 0.6))
relevancy_b <- sample(likert_ordinals, 72, replace=TRUE, prob=c(0.0, 0.05, 0.1, 0.25, 0.6))
empty_relevancy <- as.integer(runif(8, min=0, max=72))
relevancy_a[empty_relevancy] <- NA
relevancy_b[empty_relevancy] <- NA

# Original evaluation data
# question_a <- c()
# question_b <- c()
# codelist_a <- c()
# codelist_b <- c()
# name_a <- c()
# name_b <- c()
# datatype_a <- c()
# datatype_b <- c()
# length_a <- c()
# length_b <- c()
# description_a <- c()
# description_b <- c()
# alias_a <- c()
# alias_b <- c()
# rangechecks_a <- c()
# rangechecks_b <- c()
# goodmatch_a <- c()
# goodmatch_b <- c()
# relevancy_a <- c()
# relevancy_b <- c()

# Analysis
## Create counts for each rater, category, and rating
count(question_a, 'question_a'); count(question_b, 'question_b')
count(codelist_a, 'codelist_a'); count(codelist_b, 'codelist_b')
count(name_a, 'name_a'); count(name_b, 'name_b')
count(datatype_a, 'datatype_a'); count(datatype_b, 'datatype_b')
count(length_a, 'length_a'); count(length_b, 'length_b')
count(description_a, 'description_a'); count(description_b, 'description_b')
count(alias_a, 'alias_a'); count(alias_b, 'alias_b')
count(rangechecks_a, 'rangechecks_a'); count(rangechecks_b, 'rangechecks_b')
count(goodmatch_a, 'goodmatch_a'); count(goodmatch_b, 'goodmatch_b')
count(relevancy_a, 'relevancy_a'); count(relevancy_b, 'relevancy_b')
total_a <- c(question_a, codelist_a, name_a, datatype_a, length_a, description_a, alias_a, rangechecks_a, goodmatch_a, relevancy_a)
total_b <- c(question_b, codelist_b, name_b, datatype_b, length_b, description_b, alias_b, rangechecks_b, goodmatch_b, relevancy_b)
count(total_a, 'total_a')
count(total_b, 'total_b')

## Calcualte median for each rater and category
### Use numeric values for liketz_ordinals for median to use the native median function even though categories are ordinal.
medianAsNumericWithoutNA<-function(x) {
  median(as.numeric(x)[which(!is.na(x))])
}
medianAsNumericWithoutNA(question_a); medianAsNumericWithoutNA(question_b);
medianAsNumericWithoutNA(codelist_a); medianAsNumericWithoutNA(codelist_b);
medianAsNumericWithoutNA(name_a); medianAsNumericWithoutNA(name_b);
medianAsNumericWithoutNA(datatype_a); medianAsNumericWithoutNA(datatype_b);
medianAsNumericWithoutNA(length_a); medianAsNumericWithoutNA(length_b);
medianAsNumericWithoutNA(description_a); medianAsNumericWithoutNA(description_b);
medianAsNumericWithoutNA(alias_a); medianAsNumericWithoutNA(alias_b);
medianAsNumericWithoutNA(rangechecks_a); medianAsNumericWithoutNA(rangechecks_b);
medianAsNumericWithoutNA(goodmatch_a); medianAsNumericWithoutNA(goodmatch_b);
medianAsNumericWithoutNA(relevancy_a); medianAsNumericWithoutNA(relevancy_b);
medianAsNumericWithoutNA(total_a); medianAsNumericWithoutNA(total_b);

## Calculate median for both raters combined for each concept and category
### Use numeric values for liketz_ordinals for median to use the native median function even though categories are ordinal.
question_a_concepts <- split(question_a, (0:71) %% 24); question_b_concepts <- split(question_a, (0:71) %% 24);
question_concepts <- mapply(c, question_a_concepts, question_b_concepts, SIMPLIFY=FALSE)
question_concepts_median <- lapply(question_concepts, medianAsNumericWithoutNA)

codelist_a_concepts <- split(codelist_a, (0:71) %% 24); codelist_b_concepts <- split(codelist_b, (0:71) %% 24);
codelist_concepts <- mapply(c, codelist_a_concepts, codelist_b_concepts, SIMPLIFY=FALSE)
codelist_concepts_median <- lapply(codelist_concepts, medianAsNumericWithoutNA)
```

```
name_a_concepts <- split(name_a, (0:71) %% 24); name_b_concepts <- split(name_b, (0:71) %% 24);
name_concepts <- mapply(c, name_a_concepts, name_b_concepts, SIMPLIFY=FALSE)
name_concepts_median <- lapply(name_concepts, medianAsNumericWithoutNA)

datatype_a_concepts <- split(datatype_a, (0:71) %% 24); datatype_b_concepts <- split(datatype_b, (0:71) %% 24);
datatype_concepts <- mapply(c, datatype_a_concepts, datatype_b_concepts, SIMPLIFY=FALSE)
datatype_concepts_median <- lapply(datatype_concepts, medianAsNumericWithoutNA)

length_a_concepts <- split(length_a, (0:71) %% 24); length_b_concepts <- split(length_b, (0:71) %% 24);
length_concepts <- mapply(c, length_a_concepts, length_b_concepts, SIMPLIFY=FALSE)
length_concepts_median <- lapply(length_concepts, medianAsNumericWithoutNA)

description_a_concepts <- split(description_a, (0:71) %% 24); description_b_concepts <- split(description_b, (0:71) %% 24);
description_concepts <- mapply(c, description_a_concepts, description_b_concepts, SIMPLIFY=FALSE)
description_concepts_median <- lapply(description_concepts, medianAsNumericWithoutNA)

alias_a_concepts <- split(alias_a, (0:71) %% 24); alias_b_concepts <- split(alias_b, (0:71) %% 24);
alias_concepts <- mapply(c, alias_a_concepts, alias_b_concepts, SIMPLIFY=FALSE)
alias_concepts_median <- lapply(alias_concepts, medianAsNumericWithoutNA)

rangechecks_a_concepts <- split(rangechecks_a, (0:71) %% 24); rangechecks_b_concepts <- split(rangechecks_b, (0:71) %% 24);
rangechecks_concepts <- mapply(c, rangechecks_a_concepts, rangechecks_b_concepts, SIMPLIFY=FALSE)
rangechecks_concepts_median <- lapply(rangechecks_concepts, medianAsNumericWithoutNA)

goodmatch_a_concepts <- split(goodmatch_a, (0:71) %% 24); goodmatch_b_concepts <- split(goodmatch_b, (0:71) %% 24);
goodmatch_concepts <- mapply(c, goodmatch_a_concepts, goodmatch_b_concepts, SIMPLIFY=FALSE)
goodmatch_concepts_median <- lapply(goodmatch_concepts, medianAsNumericWithoutNA)

relevancy_a_concepts <- split(relevancy_a, (0:71) %% 24); relevancy_b_concepts <- split(relevancy_b, (0:71) %% 24);
relevancy_concepts <- mapply(c, relevancy_a_concepts, relevancy_b_concepts, SIMPLIFY=FALSE)
relevancy_concepts_median <- lapply(relevancy_concepts, medianAsNumericWithoutNA)

### Aggregate all medians of all concepts into a single matrix
concepts_median_matrix <- matrix(unlist(c(question_concepts_median, codelist_concepts_median, name_concepts_median,
datatype_concepts_median,
                              length_concepts_median, description_concepts_median, alias_concepts_median, rangechecks_concepts_median,
                              goodmatch_concepts_median, relevancy_concepts_median)), ncol=24, byrow=TRUE)

### Plot matrix as bwr heatmap
heatmap.2(concepts_median_matrix, col=bluered, dendrogram="none", Rowv=FALSE, Colv=FALSE, density.info="none", key=TRUE,
trace="none", na.color="black",
      breaks=c(0, 0.5, 1, 1.5, 2, 2.5, 3, 3.5, 4, 4.5, 5), main="Category rating median for item concepts", xlab="Concepts", ylab="Category")

## Calculate interrater agreement
### Generate contingency matrix and visualize it as heatmap to get an overview of the interrater agreement
rater_a <-list(unlist(c(question_a, codelist_a, name_a, datatype_a, length_a, description_a, alias_a, rangechecks_a, goodmatch_a, rele-
vancy_a)))
rater_b <- list(unlist(c(question_b, codelist_b, name_b, datatype_b, length_b, description_b, alias_b, rangechecks_b, goodmatch_b, rele-
vancy_b)))
ratings_table <- do.call(rbind, Map(data.frame, A=rater_a, B=rater_b))
ratings_contingency_table <- table(ratings_table)
ratings_contingency_table

rating_pairs <- mapply(list, unlist(rater_a), unlist(rater_b), SIMPLIFY=F)
bryw <- colorRampPalette(c("black", "red", "yellow", "white"), space="rgb")(256)
heatmap.2(ratings_contingency_table, col=bryw, dendrogram="none", Rowv=FALSE, Colv=FALSE, density.info="none", key=TRUE,
trace="none", na.color="black",
      main="Interrater agreement", xlab="Rater A", ylab="Rater B")

### Calculate Krippendorff's alpha to obtain a measurement for the interrater agreement
rater_matrix <- matrix(unlist(c(rater_a, rater_b)), ncol=72*10, byrow=TRUE)
#### Calculate Krippendorff's alpha with confidence intervals using default parameters from paper
#### "Measuring inter-rater reliability for nominal data – which coefficients and confidence intervals are appropriate?"
k_alpha(t(rater_matrix), alpha_q=0.05, nboot=1000, scaling="ordinal")

#### Calculate Krippendorff's alpha from irr package only as a control value to th above calculation
kripp.alpha(rater_matrix, method="ordinal")
```
