# Supplementary material for: Pragmatic MDR: a metadata repository with bottom-up standardization of medical metadata through reuse
Source: BMC Med Inform Decis Mak. 2021 May 17;21:160. doi: 10.1186/s12911-021-01524-8 (PMC8130274; doi:10.1186/s12911-021-01524-8)
Supplement: Supplementary file 3 — Additional file 3. Table with example requests for the REST API of the pragmatic MDR proof of concept. [file 12911_2021_1524_MOESM3_ESM.pdf]

Additional file 3: Table with example requests and results for the REST API of the pragmatic MDR proof of concept application.

**Request**	**Results**
/items/8946	Query the item resource with id 8946.
/items?query=age	Find all items resources that contain "age" with the default sorting that combines the matching and number of occurrences of the resource.
/items?query=age&sort=occurrences,desc	Find items with "age" and sort descending by number of occurrences,
/items?size=20&page=2	Request the third page with 20 results of all item resources.
/items?query=name:age+AND+surg*	Find items that contain the prefix "surg" and have a name with "age".
