# Supplementary material for: Pragmatic MDR: a metadata repository with bottom-up standardization of medical metadata through reuse
Source: BMC Med Inform Decis Mak. 2021 May 17;21:160. doi: 10.1186/s12911-021-01524-8 (PMC8130274; doi:10.1186/s12911-021-01524-8)
Supplement: Supplementary file 4 — Additional file 4. Overview of item definitions for the quality evaluation of bottom-up standards. [file 12911_2021_1524_MOESM4_ESM.pdf]

Additional file 4: Overview of item definitions for quality evaluation of bottom-up standards.

Group	Concept #	Item Concept Query	Item #	Top three search results in pragmatic MDR	Occ.
CDASH vital signs	1	Body Height (total results: 4793)	1	Body height	21
			2	Body Height	21
			3	Body Height	11
	2	Body Weight (total results: 6134)	4	Body weight	40
			5	Body weight	19
			6	Body weight	21
	3	Diastolic BP (total results: 3062)	7	BP:	13
			8	Semi-supine BP Diastolic	10
			9	Standing BP Diastolic	10
	4	Systolic BP (total results: 3361)	10	BP:	13
			11	Systolic BP	1
			12	Semi-supine BP Systolic	10
	5	Pulse (total results: 1285)	13	Pulse (bpm)	94
			14	Pulse	28
			15	Pulse	19
	6	Body Temperature (total results: 5001)	16	Body temperature	22
			17	Body Temperature	9
			18	Body Temperature	6
Most frequent LOINC codes	7	Creatinine (total results: 3495)	19	Creatinine	5
			20	Creatinine	18
			21	Creatinine Clearance	5
	8	Hemoglobin (total results: 2190)	22	Hemoglobin	29
			23	Hemoglobin	7
			24	Hemoglobin	14
	9	Potassium (total results: 739)	25	Potassium	30
			26	Potassium	13
			27	Potassium	12
	10	Glucose (total results: 1808)	28	Glucose	30
			29	Glucose	14
			30	Glucose	11
	11	Sodium (total results: 673)	31	Sodium	30
			32	Sodium	20
			33	Sodium	13
	12	Urea nitrogen (total results: 664)	34	Blood urea nitrogen	10
			35	Serum Urea	1
			36	Serum Urea Nitrogen	1
Most frequent ischaemic heart disease related UMLS concepts from MDM Portal	13	Myocardial Infarction (total results: 2838)	37	Myocardial infarction	3
			38	Myocardial Infarction	21
			39	Myocardial infarction	10
	14	Coronary Artery Bypass Surgery (total results: 7887)	40	CABG	1
			41	Coronary artery bypass surgery (CABG-Op)	1
			42	Coronary Artery Bypass Surgery	2
	15	Angina Pectoris (total results: 1297)	43	Angina pectoris	8
			44	Angina	1
			45	Angina Pectoris	2
	16	Myocardial Ischemia (total results: 2868)	46	evidence of myocardial ischemia	1
			47	exercise-induced myocardial ischemia	1
			48	5. Other interventions: [6] ECG	1
	17	Coronary heart disease (total results: 27580)	49	Any contraindication to the use of Adrenaline	1
			50	Coronary Artery Disease (heart disease)	2
			51	coronary heart disease	2
	18	Coronary revascularization (total results: 2692)	52	[4] Non-coronary revascularisation	1
			53	4. Has the subject undergone a coronary	1
			54	Time of revascularisation percutaneous coronary	1
Most frequent stroke related UMLS concepts from MDM Portal	19	Cerebrovascular accident (total results: 1236)	55	Cerebrovascular Accident	21
			56	Cerebrovascular accident within one yar	1
			57	Cerebrovascular disease	1
	20	Hemorrhage (total results: 814)	58	Is a vitreous hemorrhage present?	30
			59	Hemorrhage	1
			60	Retinal Hemorrhage	4
	21	Transient Ischemic Attack (total results: 1227)	61	History of transient ischemic attack (TIA)	2
			62	Transient Ischemic Attack	21
			63	Stroke/transient ischemic attack (TIA)	1
	22	Muscle Weakness (total results: 922)	64	Muscle weakness	2
			65	39. Muscle weakness	1
			66	Musculoskeletal Muscle atrophy or weakness	1
	23	grip strength test left hand (total results: 13963)	67	Left Grip Strength Max-Grip Test 1	2
			68	Left Grip Strength Max-Grip Test 1	2
			69	Left Grip Strength Max-Grip Test 2	2
	24	Dysarthria (total results: 126)	70	Dysarthria	4
			71	Dysarthria	1
			72	Dysarthria	1

For the quality evaluation of bottom-up standards we used the same proceeding to determine relevant data elements as for the standardization evaluation. So, we refer to the main manuscript for a detailed description. Note that this analysis was performed directly after the initial synchronization, so that it is based on fewer content than the standardization evaluation, which was performed one year later.
