# Supplementary figures and images for: Pragmatic MDR: a metadata repository with bottom-up standardization of medical metadata through reuse
Source: BMC Med Inform Decis Mak. 2021 May 17;21:160. doi: 10.1186/s12911-021-01524-8 (PMC8130274; doi:10.1186/s12911-021-01524-8)

Additional file 5: Contingency table and heat map for rater agreement.

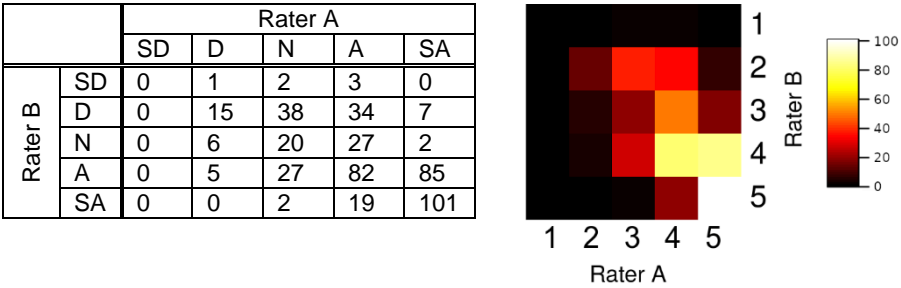

Supplement: Supplementary file 5 — Additional file 5. Contingency table and heat map for rater agreement. [file 12911_2021_1524_MOESM5_ESM.pdf]
