# Supplementary material for: Pragmatic MDR: a metadata repository with bottom-up standardization of medical metadata through reuse
Source: BMC Med Inform Decis Mak. 2021 May 17;21:160. doi: 10.1186/s12911-021-01524-8 (PMC8130274; doi:10.1186/s12911-021-01524-8)
Supplement: Supplementary file 6 — Additional file 6. Heat map for median ratings for each item concept across both raters and top three search results. [file 12911_2021_1524_MOESM6_ESM.pdf]

Additional file 6: Heat map for median ratings for each item concept across both raters and top three search results.

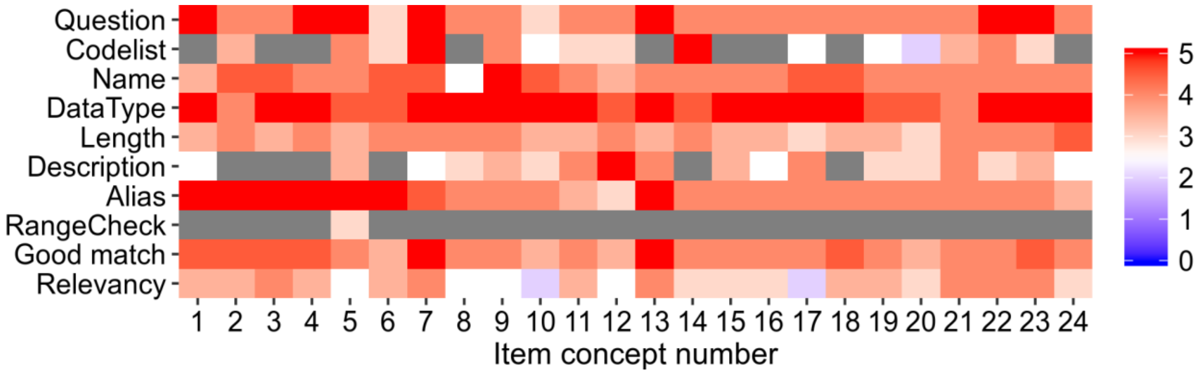

Visualization of median ratings for each item concept across both raters and the top three search results as heat map. Negative ratings are blue, neutral white, and positive ratings are red. Gray indicates that no median could be determined due to undefined properties.
